# A deep learning model of tumor cell architecture elucidates response and resistance to CDK4/6 inhibitors

**DOI:** 10.1038/s43018-024-00740-1

**Published:** 2024-03-05

**Authors:** Sungjoon Park, Erica Silva, Akshat Singhal, Marcus R Kelly, Kate Licon, Isabella Panagiotou, Catalina Fogg, Samson Fong, John J. Y. Lee, Xiaoyu Zhao, Robin Bachelder, Barbara A. Parker, Kay T. Yeung, Trey Ideker

**Affiliations:** 1 Department of Medicine, University of California, San Diego, La Jolla, CA, USA.; 2 Program in Biomedical Sciences, University of California, San Diego, La Jolla, CA, USA.; 3 Department of Computer Science and Engineering, University of California, San Diego, La Jolla, CA, USA.; 4 Moores Cancer Center, University of California, San Diego, San Diego, CA, USA.; 5 Department of Bioengineering, University of California, San Diego, La Jolla, CA, USA.; 6 These authors contributed equally: Sungjoon Park, Erica Silva, Akshat Singhal.

## Abstract

Cyclin-dependent kinase 4 and 6 inhibitors (CDK4/6is) have revolutionized breast cancer therapy. However, <50% of patients have an objective response, and nearly all patients develop resistance during therapy. To elucidate the underlying mechanisms, we constructed an interpretable deep learning model of the response to palbociclib, a CDK4/6i, based on a reference map of multiprotein assemblies in cancer. The model identifies eight core assemblies that integrate rare and common alterations across 90 genes to stratify palbociclib-sensitive versus palbociclib-resistant cell lines. Predictions translate to patients and patient-derived xenografts, whereas single-gene biomarkers do not. Most predictive assemblies can be shown by CRISPR–Cas9 genetic disruption to regulate the CDK4/6i response. Validated assemblies relate to cell-cycle control, growth factor signaling and a histone regulatory complex that we show promotes S-phase entry through the activation of the histone modifiers KAT6A and TBL1XR1 and the transcription factor RUNX1. This study enables an integrated assessment of how a tumor’s genetic profile modulates CDK4/6i resistance.

Cell-cycle activation and sustained proliferation are hallmarks of cancer^1^. Cyclin-dependent kinases 4 and 6 (CDK4/6) trigger cells to pass the G_1_/S cell-cycle restriction point by phosphorylating the retinoblastoma (RB) transcriptional repressor and its paralogs. Inhibiting these kinases has been of high interest in cancer drug development^2,3^. Thus far, three CDK4/6 inhibitors (CDK4/6is) (palbociclib, ribociclib and abemaciclib) have been approved in combination with endocrine therapy for the treatment of hormone receptor-positive, human epidermal growth factor (EGF) receptor 2-negative (HR^+^, HER2^−^) breast cancer, and clinical trials are underway in a spectrum of other tissue types^4^. In metastatic breast cancer, these agents have appreciably improved treatment outcomes, increasing progression-free and overall survival with manageable adverse effects^4,5^. However, objective tumor response is observed in <50% of patients who receive CDK4/6 inhibitors as first-line therapy, and nearly all initially responsive patients develop drug resistance with subsequent mortality^6,7^.

Studies of resistance to these drugs have largely defined two groups of molecular biomarkers: loss-of-function alterations to anti-proliferative CDK pathway genes (for example, *CDKN2A/B/C* or *RB1*) or gain-of-function alterations to progrowth genes (for example, *CDK2*, *CDK4/6*, *CCND1*, *CCNE1*, *E2F* or *PIK3CA*). These markers have been characterized predominantly in preclinical in vitro studies, with clinical assessments obtained primarily through retrospective analyses^4,5^. *RB1* mutation bears the strongest burden of evidence, as it has been extensively associated with CDK4/6 drug resistance in cell lines and patient cohorts^8^. However, it and other markers have met with inconsistent results in prospective clinical trials^8^, suggesting that our understanding of this drug response is still incomplete.

Deep learning is a powerful general methodology in precision medicine, including the use of molecular profiles to predict drug responses^9^. Such models are typically trained to maximize the accuracy of outcome prediction (for example, whether a patient will respond to a drug) without attempting to reveal the internal cellular and molecular mechanisms by which that outcome is achieved. In this regard, it is notoriously difficult to interpret which molecular features are relevant and even more difficult to describe how these features integrate with one another in the logic of molecular pathways^10^. To create models that are both predictive and interpretable^11,12^, we and others have advanced a series of ‘visible’ neural network (VNN) architectures^13–16^ that are guided by knowledge maps of cellular components and functions. For example, using such a model, Elmarakeby et al. found that metastatic outcomes in prostate cancer were well predicted by convergent genetic alterations within a mouse double minute (MDM)–tumor protein 53 (TP53) inhibition pathway, implicating *MDM4* in resistance to antiandrogen therapy^15^.

Thus far, VNN models have been structured using Gene Ontology^17^ or Reactome^18^, two general human expert-curated databases of known cellular components and functions that have not been explicitly designed to capture the molecular pathways of cancer. To define and discover cancer mechanisms systematically, we recently developed a hierarchical map of multiprotein assemblies called NeST (Nested Systems in Tumors)^19^. To build this map, we used affinity purification–mass spectrometry (AP–MS) to interrogate the physical interactions of a broad set of frequently altered cancer proteins. These data were integrated with other systematic omics datasets to create a large cancer protein–protein association network. Structural analysis of this network revealed a hierarchy of protein assemblies in which small, specific complexes nest within larger communities corresponding to broad processes and organelles. NeST was defined as the final hierarchy of 395 assemblies found to be under significant selection pressure for somatic mutations in one or more adult tumor types (Fig. 1a)^19^. Beyond the identification of the mutated protein assemblies, NeST has not yet been used to inform drug response models.

Here, we use this experimentally derived NeST map as the foundation for a visible deep learning approach to understand how patterns of genetic alterations govern the tumor response to CDK4/6 inhibition. This model is functionally predictive of palbociclib treatment outcomes and can be structurally interpreted, revealing a focal set of protein assemblies on which common and rare cancer mutations converge to affect drug resistance or sensitivity (Fig. 1b).

## Results

### Implementation of a cancer-oriented VNN

**Extended Data Fig. 1 |:**
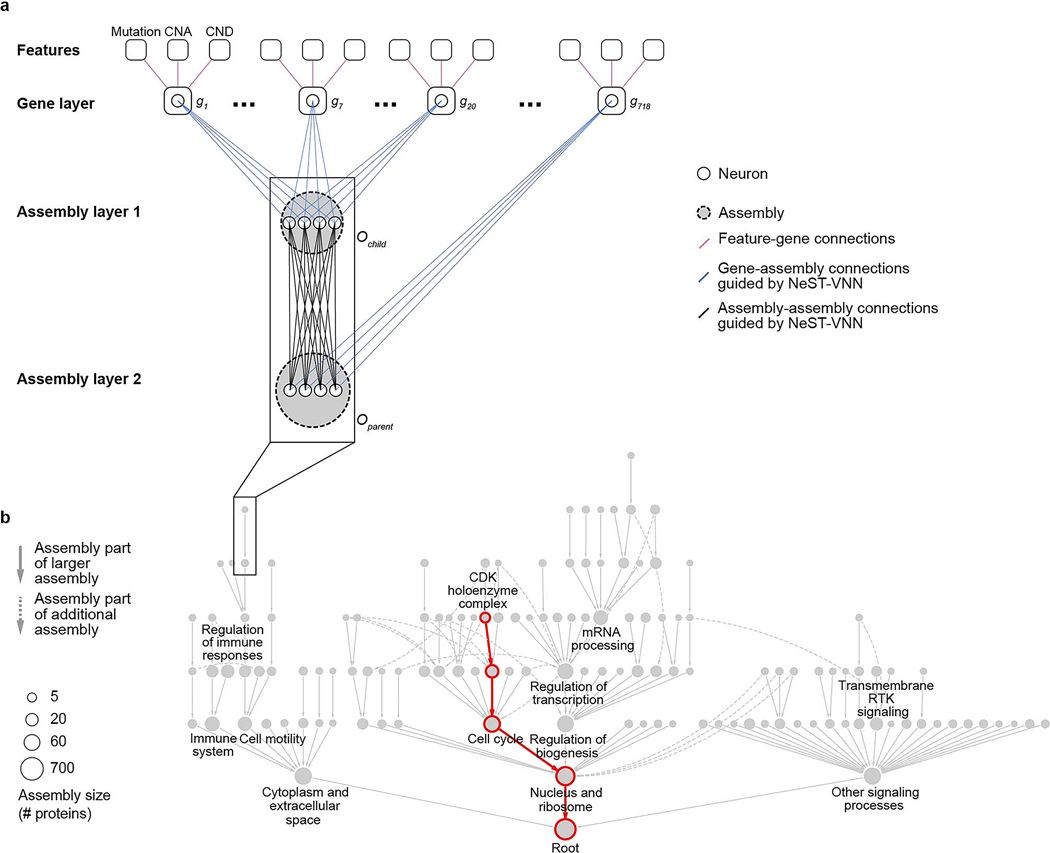
NeST-VNN schematic. **a**, The first layer of NeST-VNN incorporates gene-level features, including gene mutations, copy number amplifications (CNA), and copy number deletions (CND). Subsequent assembly layers aggregate gene-level features into assembly-level information, guided by the hierarchical relationships defined by the NeST map. The output state of each gene (*g*) and assembly (*O*) is represented by artificial neurons (one neuron per gene, multiple neurons per assembly). **b**, Position of the assemblies detailed in panel **a** within the greater NeST map. Each node indicates a protein assembly. An example path of information flow, from the neurons of CDK holoenzyme complex to Cell cycle through to the model Root, is shown in red.

**Fig. 1 |:**
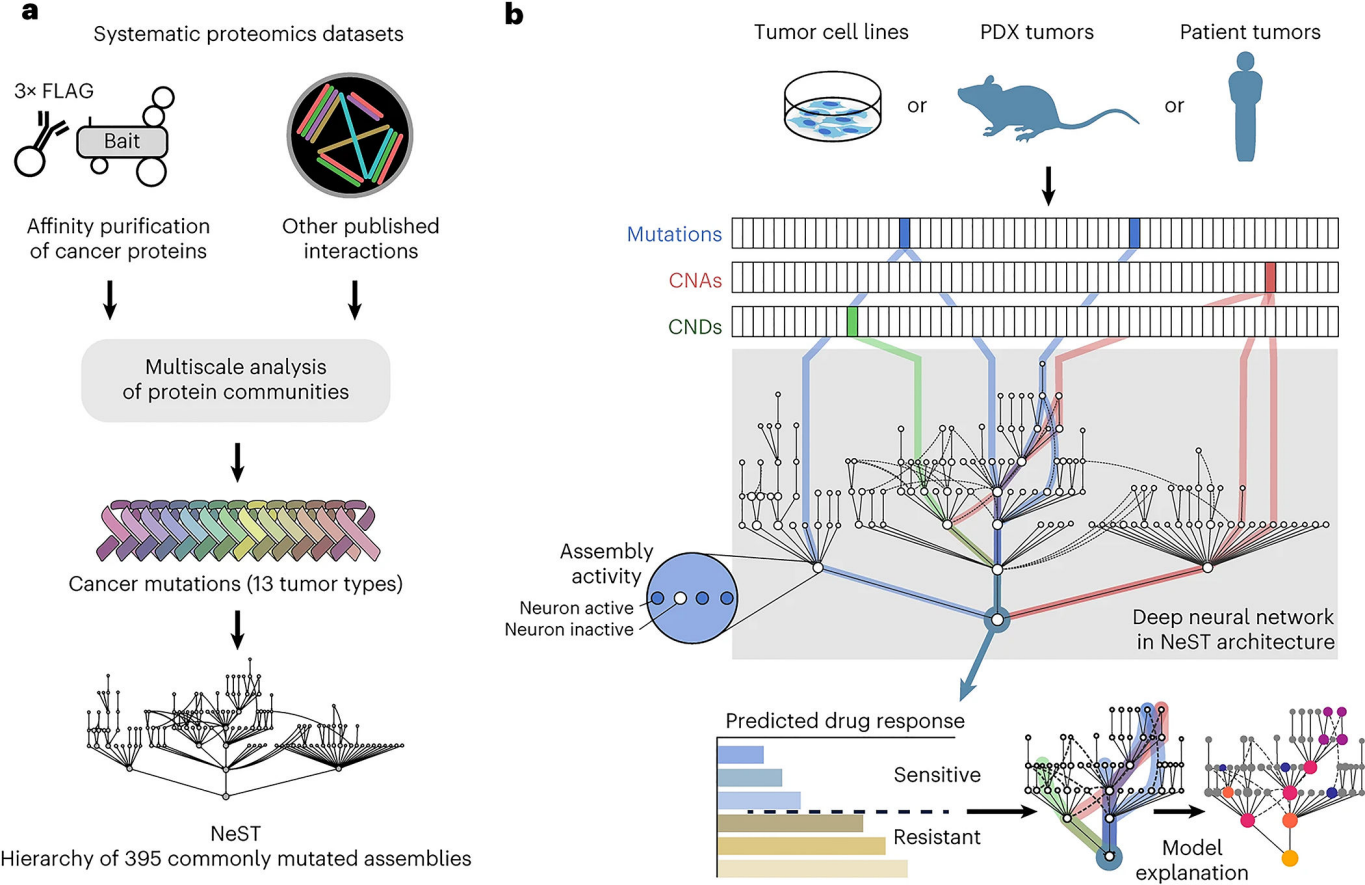
Architecture and features of the visible deep learning model. **a**, Workflow depicting the construction of the NeST hierarchy of cancer protein assemblies by Zheng et al.^19^. AP–MS data for 61 cancer protein baits were combined with a compendium of other systematic proteomics and omics datasets to produce an integrated protein network. This network was analyzed by multiscale community detection to identify a hierarchy of nested protein assemblies. Those assemblies under mutational selection pressure in different tumor types were then identified, yielding the NeST map. **b**, VNN architecture for translating tumor genetic alterations (top) to drug responses (bottom) by genetic flow through the NeST map (middle). NeST is reduced to the 131 assemblies that involve genes measured by clinical gene panels (see text).

We defined a set of 718 genes assessed by one or more clinical cancer gene panels and studies, including the FoundationOne CDx panel^20^, Tempus xT^21^ and Project GENIE (Genomics Evidence Neoplasia Information Exchange)^22^. We then queried NeST to extract a hierarchy of 131 protein assemblies containing proteins encoded by the clinically assessed genes. This hierarchy was used to guide the architecture of a VNN following a previously described approach^13^ (Fig. 1b, Extended Data Fig. 1a and Methods). This model of cancer cell structure and response, which we call NeST-VNN, allowed for three binary input features per gene, describing the presence or absence of point mutation/insertion/deletion, copy number amplification (CNA) or copy number deletion (CND) (Fig. 1b and Methods). These gene-level input features were integrated within their respective protein assemblies in subsequent layers of the NeST-VNN, with each assembly represented by a bank of artificial neurons, reflecting its biological state or ‘in silico activity’ (Extended Data Fig. 1a). Connections were also established between the neurons of an assembly and those of larger assemblies that contain it (Extended Data Fig. 1b), allowing for the flow of genetic information from small focal assemblies (for example, ‘CDK holoenzyme complex’) to larger-scale assemblies and superassemblies (for example, ‘cell cycle’). The final protein assembly at the root of the hierarchy represented the model output—the predicted drug response of a tumor sample given the input set of genetic alterations (Fig. 1b).

To train NeST-VNN, we leveraged drug response data for 1,244 genomically characterized tumor cell lines^23^, obtained by harmonizing the Cancer Therapeutics Response Portal (CTRP)^24,25^ and Genomics of Drug Sensitivity in Cancer (GDSC)^26,27^ databases (Methods). These data included the response to the CDK4/6i palbociclib, which had been well characterized in 947 cell lines. For comparative benchmarking, we also examined 50 non-CDK-related drugs investigated in at least 200 cell lines, for which the cellular responses displayed sufficient variability, with many examples of sensitivity and resistance (Methods).

### Evaluation of prediction performance

**Extended Data Fig. 2 |:**
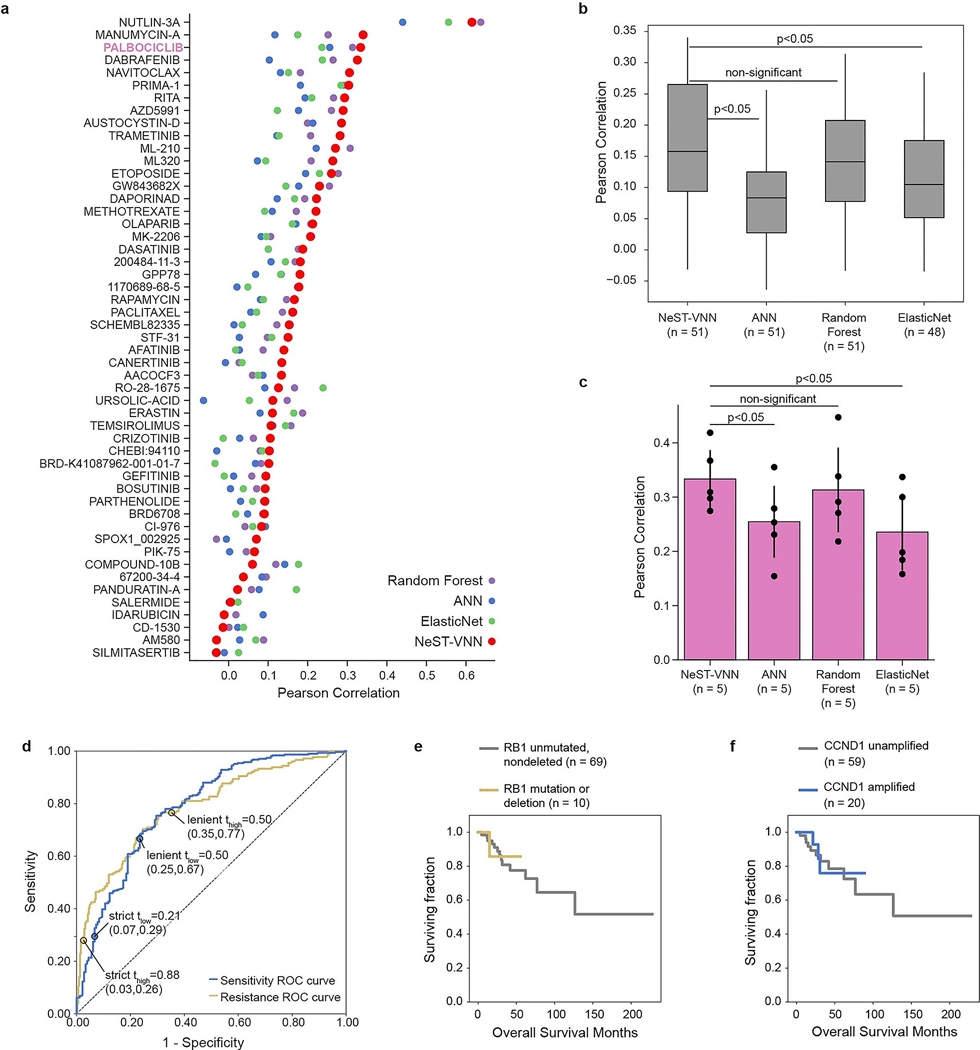
Supplemental model performance analysis. **a**, Dot plot of model performance for each of 51 drugs for NeST-VNN (red) versus 3 alternate models: ElasticNet (green), Random Forest (purple), and a conventional Artificial Neural Network (ANN, blue). Palbociclib model highlighted in pink. **b**, Boxplot of performance for all drugs. Box plots show the 25th, 50th, and 75th percentiles of Pearson correlation. P-values reflect results of a one-tailed t-test assessing whether NeST-VNN outperforms baseline models. **c**, Bar chart of performance for palbociclib models. Error bars represent 95% confidence intervals with mean as the midpoint. P-values reflect results of one-tailed t-test. **d**, ROC curves for predicting resistance (yellow) or sensitivity (blue) to palbociclib. Marked points indicate cutoffs used to label samples as “resistant” or “sensitive” at different stringencies. Values in parenthesis indicate the (x,y) coordinate. **e**, Survival curves for CDK4/6i-treated patients stratified by somatic mutations or copy number deletions in *RB1*. **f**, Survival curves for CDK4/6i-treated patients stratified by somatic copy number amplifications in *CCND1*.

We constructed NeST-VNN drug response models for palbociclib and separately for each of the 50 benchmark drugs, using standard neural network learning procedures based on backpropagation (Methods). Each model was trained to use the gene alteration profile of a cell line to predict the corresponding area under the dose–response curve (AUC). Training and performance assessment was conducted using nested fivefold cross-validation (Methods), with each fold setting aside 64% of cell lines for training, 16% for validation (used for tuning hyperparameters) and 20% for testing. Although nested cross-validation is computationally intensive, it fully insulates model testing from parameter tuning while maximizing the amount of testing that can be performed. We compared the NeST-VNN approach to three state-of-the-art alternatives: ElasticNet, random forest (RF) and a conventional black-box artificial neural network (ANN) (Extended Data Fig. 2a,b and Supplementary Table 1). The overall performance of NeST-VNN was generally comparable to that of the state-of-art models and often better, with NeST-VNN achieving the best performance for more than half of the tested drugs (62.7%; Extended Data Fig. 2a,b). NeST-VNN trained for palbociclib was one of the top-performing models, significantly outperforming the ElasticNet and ANN models and slightly, but not significantly, outperforming RF (Extended Data Fig. 2c and Supplementary Table 1).

**Fig. 2 |:**
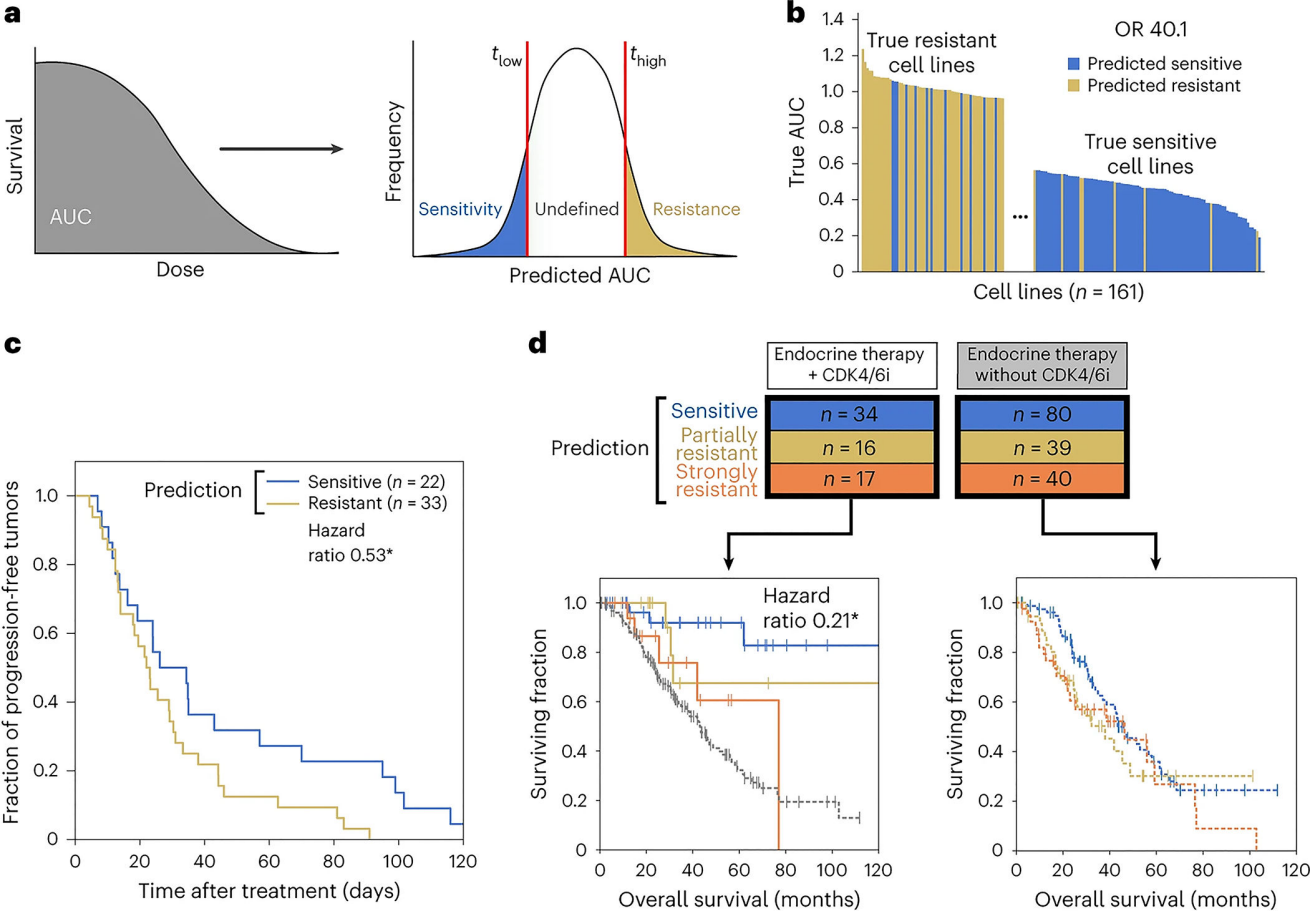
Predictive performance of the palbociclib model. **a**, Scanning cell survival to measure a continuous AUC that is thresholded to assign class labels (sensitive, undefined or resistant). **b**, Waterfall plot showing the true dose responses of tumor cell lines, with colors indicating the predicted class of each. Predicted AUC is thresholded to produce class labels (sensitive, resistant; see text). **c**, Survival curve analysis for predicting the sensitive or resistant status of PDX samples. *P < 0.05 by log-rank test. **d**, Survival curve analysis for GENIE clinical trial patients treated with CDK4/6i plus endocrine therapy (left) or endocrine therapy alone (right). Colors denote class labels for predicted CDK4/6i-sensitive (blue) and CDK4/6i-resistant (yellow/orange) patients, with additional stratification of a strongly resistant category (orange). Patients not treated with CDK4/6i therapy are shown in gray. The hazard ratio was 0.21 for strongly resistant versus sensitive predicted subgroups. *P < 0.05 by log-rank test.

To translate predictions to discrete tumor response outcomes, we thresholded the AUC such that predictions below a value *t*_low_ were labeled ‘sensitive’, those above a value *t*_high_ were labeled ‘resistant’ and those between these two thresholds were labeled ‘undefined’ (Fig. 2a). At the most inclusive setting, *t*_low_ = *t*_high_ = median(AUC), NeST-VNN could accurately discriminate between actual sensitive and resistant cell lines in heldout test data, with a diagnostic odds ratio (OR) of 6.0. Discriminative power increased substantially with more stringent thresholds. For instance, setting the thresholds 1 s.d. from the median (*t*_low_, *t*_high_ = median(AUC) ± s.d.(AUC)) yielded a very high OR of 40.1, indicating that samples predicted as resistant were approximately 40 times more likely to test as resistant than samples predicted as sensitive (Fig. 2b). The trade-off for higher accuracy was that samples were left undefined (66%), increasing specificity but decreasing sensitivity (Extended Data Fig. 2d).

### Translation to patient-derived xenografts and patients

Next, we examined the performance of NeST-VNN in a study of patient-derived xenografts (PDXs)^28^, including *n* = 172 samples treated with a CDK4/6i (ribociclib). Each PDX sample was classified as sensitive, resistant or undefined using thresholds at 1 s.d. from the median (*t*_low_, *t*_high_ = median(AUC) ± s.d.(AUC)). PDX samples predicted to be sensitive exhibited significantly longer progression-free survival (duration from the start of treatment to the doubling of tumor volume) than those predicted to be resistant, suggesting that predicted sensitivity was associated with impaired tumor growth (log-rank *P* = 0.04, hazard ratio 0.53, 95% confidence interval 0.30–0.97; Fig. 2c and Methods).

After this analysis in xenografts, we evaluated model performance in predicting treatment outcomes for 226 patients with breast cancer from the GENIE metastatic breast cancer cohort^22^. These patients had been treated with baseline endocrine therapy with (*n* = 67) or without (*n* = 159) a CDK4/6i. Each patient was classified as ‘sensitive’ or ‘resistant’ using the threshold *t*_low_ = *t*_high_ = median(AUC) (no ‘undefined’ category was used because the number of treated samples was less than that for the earlier PDX or cell line analysis). The resistant category was further equally split to denote ‘partially resistant’ and ‘strongly resistant’ subgroups. For patients treated with a CDK4/6i, those predicted to be sensitive had significantly longer survival than those predicted to be strongly resistant (log-rank *P* = 0.02, hazard ratio 0.21, 95% confidence interval 0.05–0.91; Fig. 2d). Moreover, for the strongly resistant subgroup, the addition of the CDK4/6i failed to produce a significant increase in overall survival compared to baseline therapy (*P* = 0.37). These predictions outperformed single-gene markers of palbociclib resistance^29^ (*RB1* mutation) or sensitivity^30,31^ (*CCND1* CNA) that had been previously suggested, consistent with the mixed results of these markers in clinical trials^8^ (Extended Data Fig. 2e,f). In patients who did not receive a CDK4/6i, no significant survival differences were observed among the predicted sensitive/resistant/strongly resistant class labels (all comparisons with log-rank *P* > 0.1; Fig. 2d). These results indicate that the NeST-VNN palbociclib model translates to the population of patients with breast cancer and is specifically predictive of response rather than generally prognostic of patient survival.

### Interpreting the model to identify important protein assemblies

**Extended Data Fig. 3 |:**
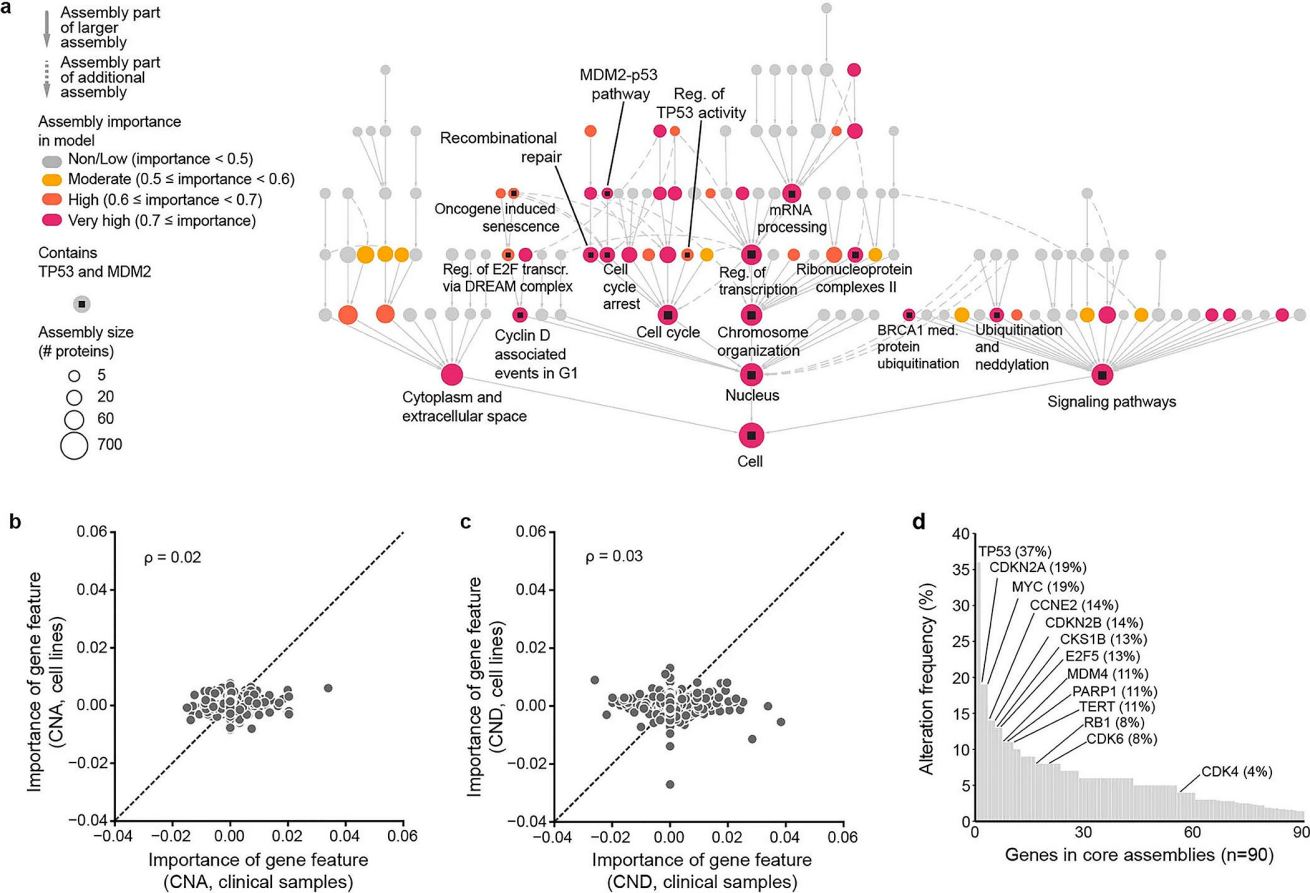
Supplemental model interpretation. **a**, NeST-VNN interpretation of the Nutlin-3a response. Nodes indicate assemblies; node sizes indicate assembly sizes in numbers of proteins; node colors indicate degrees of importance for response prediction; squares inside the nodes indicate whether the assembly contains TP53 and MDM2, drug targets of Nutlin-3a. **b**, Scatter plots of gene importance based on copy number amplifications (CNA) in clinical vs. cell line contexts (x vs. y). **c**, same as panel **b** except the gene importance is based on copy number deletions (CND). **d**, Alteration frequencies of genes within core assemblies. Includes somatic mutations, CNA, and CND observed in the TCGA/ICGC pan-cancer data.

**Fig. 3 |:**
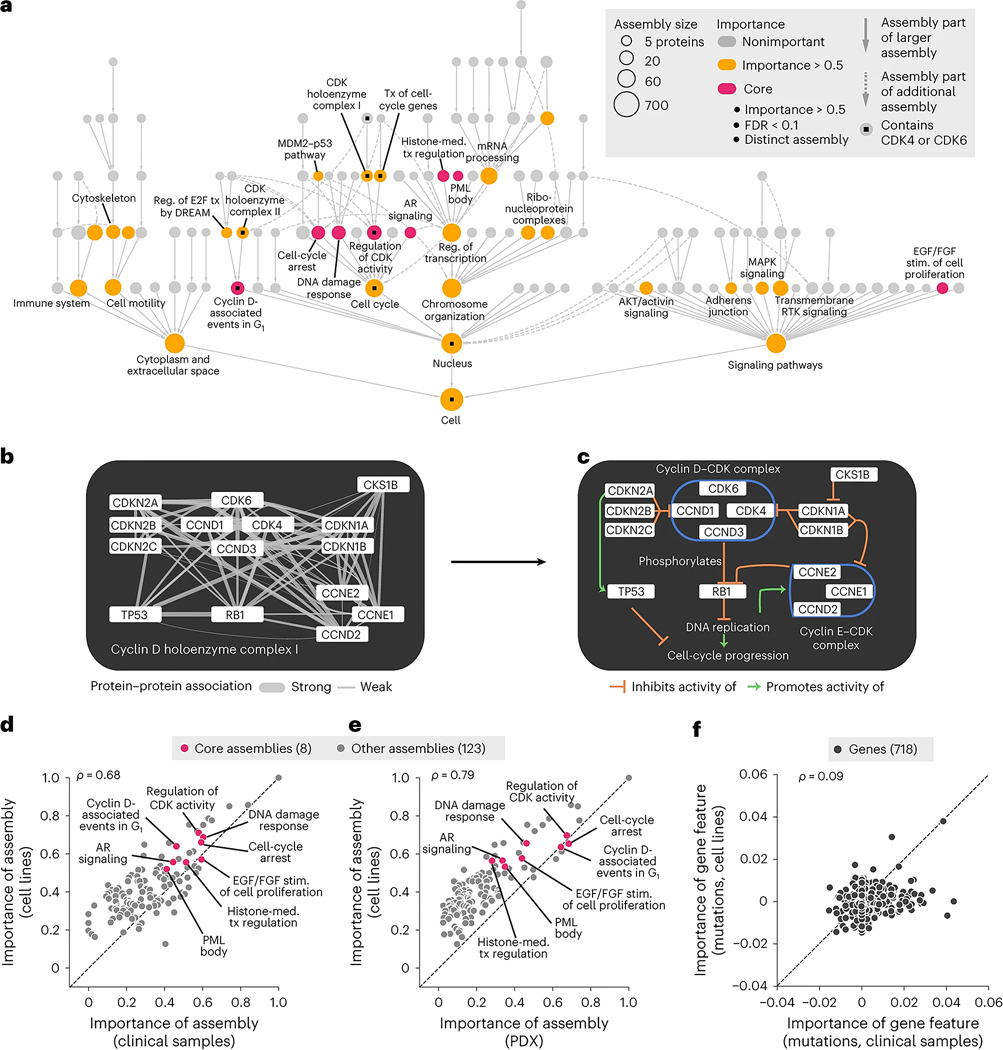
Interpretation of palbociclib response mechanisms. **a**, NeST-VNN interpretation of the palbociclib response. Nodes indicate assemblies, whereas node sizes indicate assembly sizes in numbers of proteins. Colors indicate the degree of importance for response prediction: yellow, assemblies with importance > 0.5; red, ‘core’ assemblies, which bring the additional requirement of FDR ≤ 0.1 and exclude redundant assemblies of lesser importance (Jaccard similarity > 0.5). Assemblies containing CDK4 or CDK6 are marked with small black squares. **b**, Protein interaction network defining the CDK holoenzyme complex I (NeST:110), which contains CDK4 and CDK6. Edges represent biophysical protein–protein associations, with the edge thickness reflecting the strength of the evidence for association. **c**, Diagram of known functional associations among NeST:110 proteins in the context of cell-cycle progression. The cyclin D–CDK complex inhibits RB1 by phosphorylation, such that it no longer transcriptionally represses genes required for S-phase entry and subsequent DNA replication. **d**, Scatterplot of assembly importance in the clinical versus cell line contexts (*x* axis versus *y* axis). **e**, Scatterplot of assembly importance in the PDX versus cell line contexts (*x* axis versus *y* axis). **f**, Scatterplot of gene mutation importance in the clinical versus cell line contexts (*x* axis versus *y* axis). DREAM, dimerization partner, RB-like, E2F and multivulval class B; MAPK, mitogen-activated protein kinase; RTK, receptor tyrosine kinase; Reg., regulation; tx, transcription; med., mediated; stim., stimulation.

Having seen that the NeST-VNN model was predictive of drug response in tumor cell lines, PDX samples and patients, we sought to interpret which protein assemblies were important to this process. Following a previous method^13^, we computed a quantitative importance score for each assembly according to how well its in silico activity was associated with the final drug response prediction (Fig. 3a, Supplementary Table 2 and Methods). Assemblies containing the primary CDK4 and CDK6 drug targets were of significantly higher importance than expected by chance, serving as positive controls (*P* = 5 × 10^−5^; Fig. 3a and Supplementary Table 2). For example, one of the important CDK assemblies was NeST:110 (CDK holoenzyme complex I; Fig. 3b,c), comprising the cyclin D–CDK4–CDK6 complex along with upstream inhibitors (CDKN1/2 protein families) and downstream targets (RB1). Positive control assemblies were also observed for other top-performing drug models; the model for the drug nutlin-3a, which targets TP53 activity through MDM2, placed high importance on assemblies containing these proteins (*P* = 6.8 × 10^−10^; Extended Data Figs. 2a and 3a).

For all drug models, assembly importance tended to increase with size and depth in the hierarchy, reflecting the progressive integration of genetic information. Assembly importance was similar between cell lines and patient tumors (Fig. 3d) or PDX samples (Fig. 3e). In contrast, little correlation was observed between cell lines and clinical samples when examining the importance of individual gene mutations (Fig. 3f) or copy number aberrations (Extended Data Fig. 3b,c). These results are consistent with the premise that most individual genetic alterations are rare, with variable incidence across contexts^32^, and suggest that the effects of genetic alterations on protein assemblies can be substantially more stable.

Of 33 assemblies that were of high importance for palbociclib response prediction in cell lines (importance ≥ 0.5), we focused on eight distinct minimally overlapping assemblies whose importance scores remained significant under multiple-hypothesis correction (hereafter referred to as ‘core assemblies’; Methods). Beyond regulation of CDK activity, core assemblies spanned histone and chromatin regulation, DNA damage response and growth factor signaling (Fig. 3a), integrating rare and common genetic alterations across 90 genes (Extended Data Fig. 3d). Most core assemblies were also important for predicting outcomes in clinical and PDX samples (Fig. 3d,e).

### Systematic validation of core assemblies by loss-of-function screens

**Fig. 4 |:**
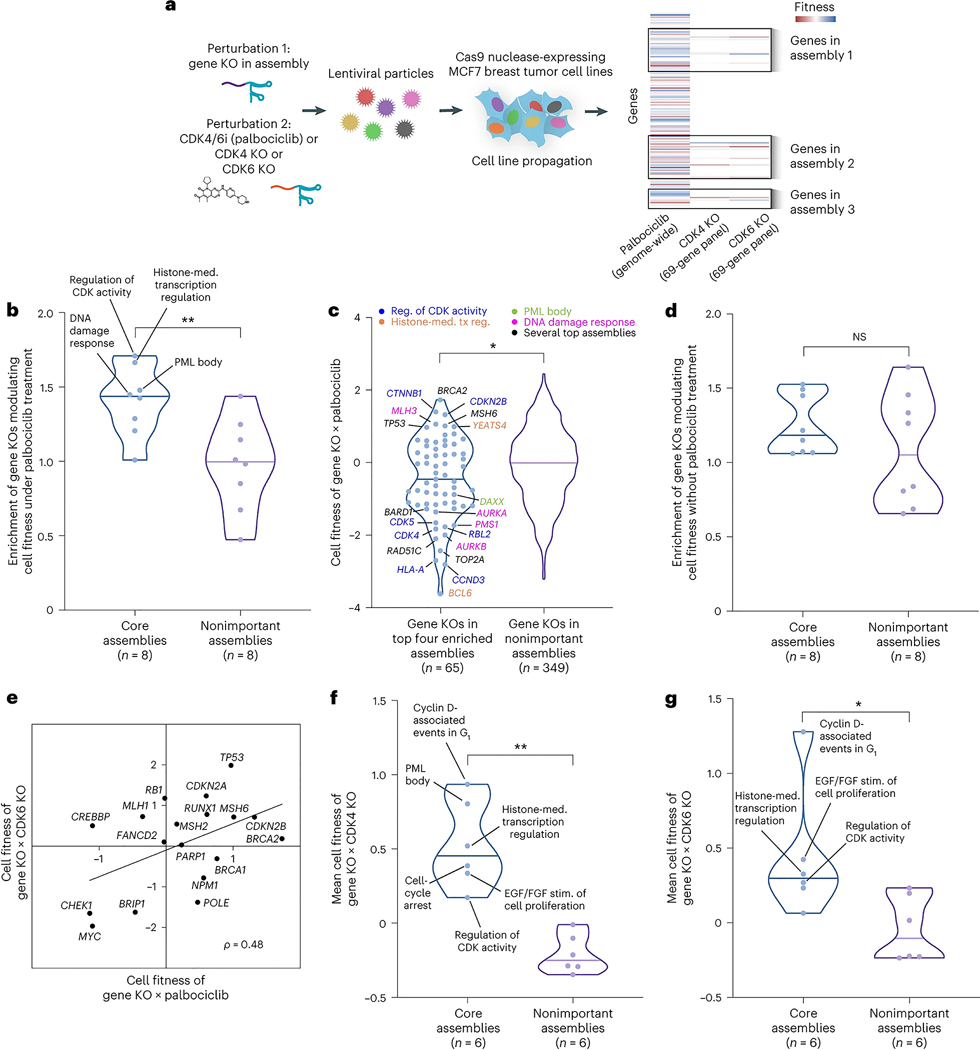
Systematic validation of palbociclib response mechanisms. **a**, Schematic overview of CRISPR screens in MCF7 breast tumor cells. Individual sgRNAs targeting genes in protein assemblies were combined with palbociclib (CDK4/6i) or a second sgRNA targeting *CDK4* or *CDK6*. Cells harboring the Cas9 nuclease were infected with lentiviral-packaged sgRNAs and propagated under selection. The palbociclib screen was from Carpintero-Fernández et al.^33^; CDK4 and CDK6 KO screens were newly generated in the present study. **b**, Violin plots illustrating the enrichment of assemblies for gene KOs modulating cell fitness in the context of palbociclib treatment, comparing core assemblies defined by NeST-VNN versus the same number of nonimportant assemblies (randomly selected among those with importance < 0.5). **P < 0.01 by one-tailed Mann–Whitney *U* test. GSEA^76^ was conducted to calculate enrichment scores. **c**, Left, violin plot illustrating the effects on cell fitness due to CRISPR KO of each gene in the top four enriched assemblies shown in **b**. Point color indicates the assembly relevant to each gene. Right, similar plot showing the effects for gene KOs in nonimportant assemblies (negative control). Cell fitness is *z* score normalized across all tested gene KOs, with *z* > 0 indicating increased fitness relative to average and *z* < 0 indicating decreased fitness. *P < 0.05 by two-tailed Mann–Whitney *U* test. **d**, Violin plots illustrating the enrichment of assemblies for gene KOs modulating cell fitness without palbociclib treatment, comparing the core assemblies versus the same number of nonimportant assemblies. NS, not significant by one-tailed Mann–Whitney *U* test. **e**, Scatterplot of cell fitness of gene KOs in the context of CDK4/6i (*x* axis) versus CDK6 KO (*y* axis). Genes shown are from the top four assemblies in **b** (*n* = 18). **f**, Violin plots illustrating the mean fitness across gene KOs in core assemblies versus the same number of gene KOs from nonimportant assemblies in combination with CDK4 KO. **P < 0.01 by two-tailed Mann–Whitney *U* test. **g**, Same as **f**, except gene KOs are combined with CDK6 KO. *P < 0.05 by two-tailed Mann–Whitney *U* test. In **f** and **g**, two core assemblies did not have sufficient coverage in the gene panel; thus, six of the eight core assemblies were tested.

We next sought to validate the palbociclib core assemblies using two CRISPR (clustered regularly interspaced short palindromic repeat) loss-of-function screens (Fig. 4a): a published chemogenetic screen involving genome-wide knockout (KO) of single genes in combination with palbociclib treatment^33^ and a de novo dual CRISPR screen in which we paired gene KOs in selected NeST-VNN assemblies with a second gene KO targeting *CDK4* or *CDK6* (Fig. 4a and Methods). For the chemogenetic screen, we assessed each assembly in NeST for the enrichment of genes whose KOs modulate cell fitness in the context of palbociclib treatment (Methods). The enrichments of the eight core assemblies tended to be significantly higher than those of nonimportant controls (*P* = 0.005, Mann–Whitney *U* test), with four of these assemblies in particular (regulation of CDK activity, histone-mediated transcription regulation, DNA damage response, promyelocytic leukemia (PML) body) showing stronger effects than any assembly in the control set (Fig. 4b and Supplementary Table 3). Such enrichment was due to KOs in a diversity of genes, including roughly a dozen with extreme loss-of-fitness phenotypes (Fig. 4c; for example, *BCL6*, *CCND3*, *CDK4*, *CDK5*, *RAD51C*, *TOP2A*, *BARD*, *AURKA*, *AURKB*) and several causing gain of fitness (for example, *BRCA2*, *CTNNB1*, *CDKN2B*, *MSH6*, *MLH3*). Enrichment was not observed for a genome-wide KO screen without palbociclib treatment^34^, indicating that at least some of the effect was due to gene–drug interactions rather than independent gene essentiality (Methods and Fig. 4d). We then moved on to our de novo dual CRISPR KO screen and noted that this screen and the earlier chemogenetic screen were reasonably consistent with respect to gene KO fitness effects (Pearson *ρ* = 0.48; Fig. 4e). Disruptions in all six of the core assemblies with sufficient coverage in our gene KO panel displayed a trend toward increased cell fitness (Fig. 4f,g and Supplementary Table 3). Taken together, these results indicate that engineered genetic disruptions in protein assemblies identified by NeST-VNN can influence tumor cell growth in the setting of CDK4/6 inhibition, whether such inhibition is induced by a drug (Fig. 4b) or CDK4/6 KO (Fig. 4f,g).

### Exploration of gain-of-function alterations in a histone transcriptional assembly

**Extended Data Fig. 4 |:**
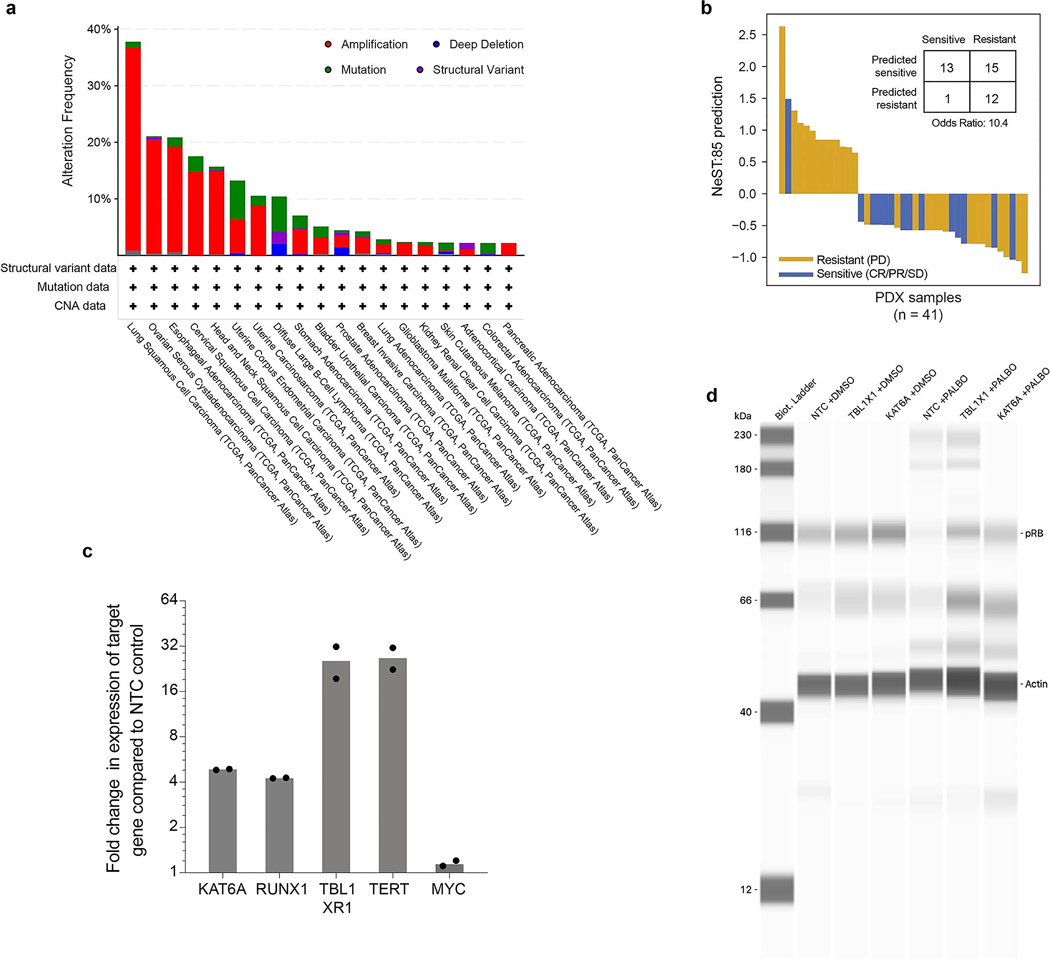
Supplemental analysis of Histone-mediated transcription regulation (NeST:85). **a**, Alteration frequency of NeST:85 genes across tumor types. Frequency (y-axis) and type (color) of genetic alterations in NeST:85 genes *KAT6A*, *MYC*, *RUNX1*, *TBL1XR1*, and *TERT*, displayed across tumor cohorts documented by the cBioPortal (x-axis). Downloaded from cbioportal.org on 14 July 2023. **b**, Waterfall plot illustrating NeST:85 prediction (y-axis) in PDX samples (x-axis, n = 41). The prediction was determined from the first principal component (PC1) of the in-silico activity of the NeST:85 assembly, thresholded (median ± stdev) to assign class labels (predicted sensitive/undefined/predicted resistant). Bar color represents true response of a PDX sample on a CDK4/6 inhibitor (ribociclib) based on the RECIST categories (yellow, resistance (PD); blue, sensitive (CR, PR, or SD)). **c**, Bar plot depicting fold increase in mRNA expression level due to overexpression of specific gene targets relative to non-targeting control (NTC). Bars indicate means of repeat experiments, with technical replicate data points shown (n = 2). **d**, Full capillary western blot image of phospho-RB (pRB) level for nominal conditions (non-targeting control, NTC), TBL1XR1 overexpression, or KAT6A overexpression. A representative image from two independent experiments.

**Fig. 5 |:**
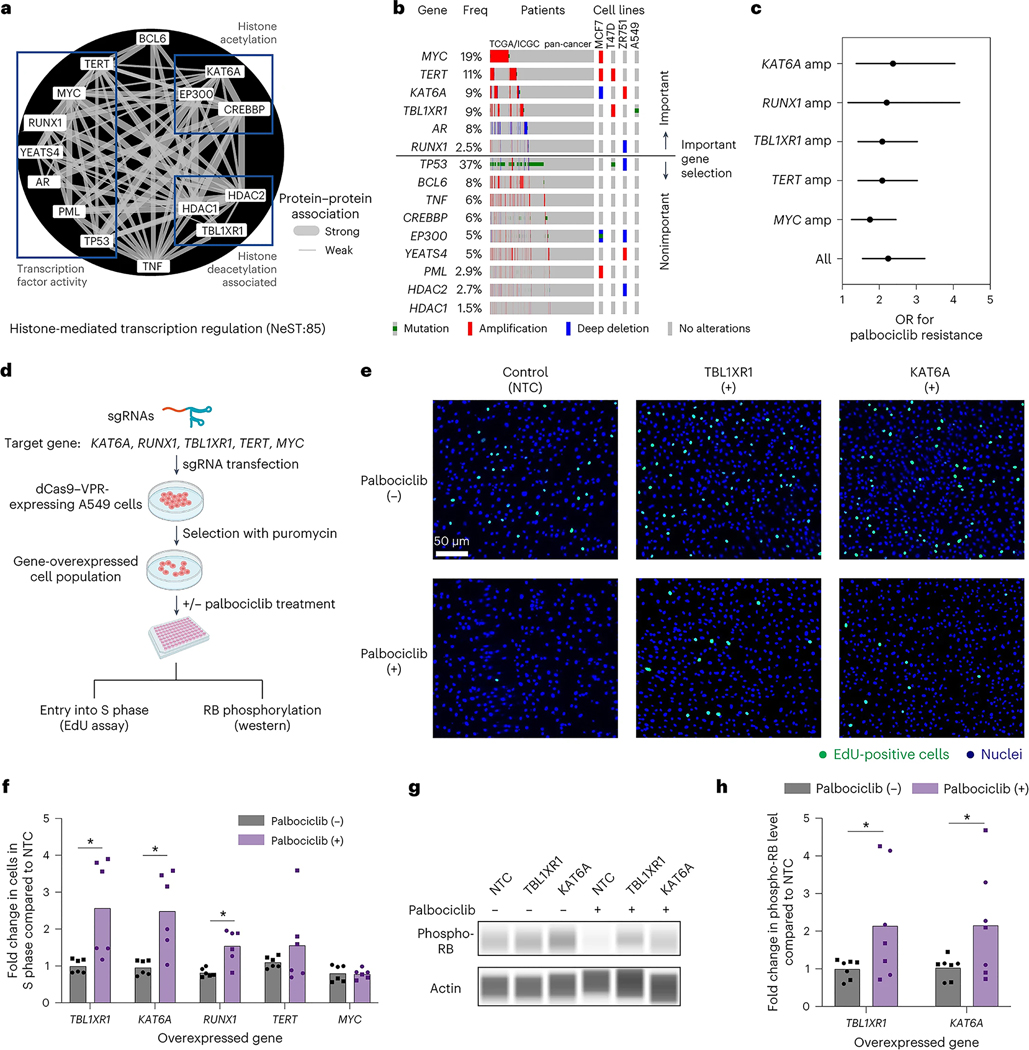
Exploring the NeST:85 histone-related assembly in the palbociclib response. **a**, Network diagram of NeST:85 depicting the histone-mediated transcription regulation assembly. Edges show protein–protein biophysical associations, with the edge thickness corresponding to the strength of the evidence for association. Three subgroups of protein functions are indicated in boxes. **b**, OncoPrint illustrating the genetic alteration patterns of NeST:85 genes (rows) in patient tumors from the TCGA/ICGC (International Cancer Genome Consortium) pan-cancer cohort (columns) along with representative cell lines (far right columns). Genes are sorted based on relative importance for drug resistance and then by alteration frequency (Freq) within each important or nonimportant group. **c**, ORs of important gene amplifications (amp) in NeST:85 with respect to palbociclib resistance in the TCGA/ICGC pan-cancer cohort. Error bars indicate the 95% confidence interval. **d**, Schematic overview of the CRISPRa gene overexpression screen. sgRNAs targeting the promoter regions of target genes were transfected into cells expressing the dCas9–VPR transcriptional activator. Effects were characterized by an EdU assay, which quantifies the number of cells undergoing active DNA synthesis, and by the phosphorylation status of RB, the molecular target of CDK4/6. Both palbociclib-treated and palbociclib-untreated conditions were examined. Created with BioRender.com. **e**, Cell microscopy images from an EdU incorporation assay for NTC (left), *TBL1XR1* overexpression (middle) or *KAT6A* overexpression (right). EdU-positive cells indicating active DNA synthesis are stained in green versus nuclei stained in blue with DAPI. Images are shown for palbociclib-untreated (top) versus palbociclib-treated (bottom) cells. **f**, Bar plot depicting the fold increase in cells undergoing active DNA synthesis (S phase) due to overexpression of specific target genes (*x* axis) relative to NTC. *P < 0.05 by two-tailed Welch’s *t* test. Bars indicate mean; error bars indicate ±standard error; individual replicates are shown. Circle points indicate biological replicate 1 (*n* technical replicates = 3), and square points indicate biological replicate 2 (*n* technical replicates = 3). **g**, Capillary western blot analysis of phospho-RB levels for NTC, *TBL1XR1* overexpression or *KAT6A* overexpression in palbociclib-treated or Palbociclib-untreated (DMSO) conditions. A representative image from two independent experiments is shown. **h**, Bar plot depicting the fold increase in relative phospho-RB level (phospho-RB/actin) for the overexpression of specific target genes (*x* axis) relative to NTC. *P < 0.05 by two-tailed Welch’s *t* test. Bars indicate mean; error bars indicate ±standard error; individual replicates are shown. Circle points indicate biological replicate 1 (*n* technical replicates = 3), and square points indicate biological replicate 2 (*n* technical replicates = 4).

An open question is how CDK4/6 and the G_1_/S transcriptional program interact with other cell functions, including upstream modulators and downstream effectors. A notable assembly in this regard was NeST:85 (histone-mediated transcription regulation), a densely connected complex of 15 proteins with roles in histone acetylation, deacetylation and transcriptional activation (Fig. 5a). This assembly was important for the CDK4/6i response in cell lines (Fig. 3a), PDX samples (Fig. 3e and Extended Data Fig. 4b) and patients (Fig. 3d). It had also been validated by CRISPR loss-of-function analysis (Fig. 4b and Supplementary Table 3). However, most of the frequent genetic alterations affecting this assembly in tumor cell lines or patients were not loss-of-function events but gene CNAs (Fig. 5b), which were especially prevalent in lung, oropharyngeal and gynecologic tumors (frequencies 15–35%; Extended Data Fig. 4a). CNAs also accounted for the top five genetic alterations in this assembly that were most predictive of palbociclib resistance, in particular those of *MYC*, *TERT*, *KAT6A*, *TBL1XR1* and *RUNX1* (Fig. 5c and Methods). Each of these amplifications had a resistance OR of approximately 2.0, indicating that cells harboring CNAs are twice as likely to exhibit resistance to palbociclib than cells without CNAs (Fig. 5c).

Motivated by these findings, we turned to the technique of CRISPR activation (CRISPRa), which uses the dCas9 (dead Cas9 endonuclease)–VPR (VP64–p65–Rta) transcriptional activator to increase expression from gene promoters targeted with CRISPR single guide RNAs (Fig. 5d). For these experiments, we selected A549 lung carcinoma epithelial cells, which harbor few genetic alterations in the NeST:85 assembly compared to many other common tumor cell models for which multiple genes are already amplified (Fig. 5b). We transfected short guide RNAs (sgRNAs) targeting *KAT6A*, *TBL1XR1*, *RUNX1*, *TERT* or *MYC* into A549 cells expressing dCas9–VPR and confirmed by qPCR that constructs exhibited substantial overexpression of the target gene compared to nontargeting control (NTC) sgRNAs (all except for *MYC*; Extended Data Fig. 4c). sgRNAs targeting *MYC* did not have a significant effect, consistent with prior reports that this gene is already highly expressed in A549 cells^35^. We used the thymidine analog 5-ethynyl-2′-deoxyuridine (EdU) to count the fraction of cells undergoing active DNA replication in the S phase. Overexpression of the histone modifiers *KAT6A* and *TBL1XR1* produced significant increases in the proportion of cells entering the S phase under palbociclib treatment compared to the untreated group (Fig. 5e,f; approximately 2.5-fold; *P* < 0.05); the transcription factor *RUNX1* also led to significant increases, albeit to a lesser degree (1.5-fold). We also examined the effects of *KAT6A* or *TBL1XR1* overexpression on the phosphorylation status of RB1, the direct target of the CDK4–CDK6–cyclin D complex. Capillary western blot analysis demonstrated that overexpression of these factors is indeed associated with a more than twofold increase in phospho-RB levels (Fig. 5g,h, Extended Data Fig. 4d and Methods). Together, these results indicate several NeST:85 genes whose overexpression serves to promote the cell cycle, supporting our earlier observation (Fig. 5c) that CNAs in these genes are predictive of palbociclib resistance.

## Discussion

CDK4/6 inhibitors are a well-studied class of drugs for which numerous candidate biomarkers have been identified^8^. Why has the prediction of CDK4/6i responses remained challenging? One reason is that markers with promise in cell lines (for example, *CCND1* amplification) do not consistently translate to patient populations^30,31^. Another is that individual genetic alterations that are clinically predictive may occur too rarely to have broad utility (for example, *RB1* deletion or mutation). A wider, more integrative analysis is needed to understand CDKi resistance fully^5,8^.

Toward this goal, NeST-VNN synthesizes both rare and common genetic events across a repertoire of drug response pathways, with the aim of facilitating a quantitative, integrated assessment of drug response. The modeling process begins with a map of tumor cell components, which is used to guide the topology of deep neural network models as they learn to translate genetic alterations to drug responses (Extended Data Fig. 1). The key subcellular assemblies of models that accurately capture drug responses in vitro and that translate to in vivo (for example, PDX) and clinical settings (Fig. 2) can be validated through directed CRISPR loss-of-function and/or activation screens (Figs. 4 and 5). Assemblies that pass this validation pipeline are a source of candidate biomarkers in downstream precision medicine applications. Alternatively, a model can be used in its entirety to produce a single resistance score integrating the mutational status of all proteins and assemblies.

NeST-VNN is based on NeST, a whole-cell map of cancer protein complexes derived from systematic proteomics data (see the ‘Structural architecture of the NeST-VNN model’ section in Methods). Previous drug response models have generally not incorporated outside knowledge of cell structure (many approaches, reviewed here^9,36^) or have modeled structure using databases of cellular components or pathways drawn from literature curation^9,14,15^. Biological insights informed by NeST-VNN are uniquely dependent on the composition of NeST, generating both strengths and limitations. One strength is that the model can incorporate information from numerous rare mutations in predicting a drug response insofar as these rare alterations aggregate to affect the activity of commonly altered protein assemblies with documented cancer relevance. A limitation is that NeST almost certainly does not include all relevant protein assemblies (false negatives), and some assemblies that are included may be imperfect or irrelevant to a given tumor population (false positives). Regardless, the NeST knowledgebase positions the precision medicine model as a dynamic entity, which can be updated either functionally with new incoming drug response data or structurally as NeST (or another future map) is improved by additional data. These new data need not be limited to AP–MS experiments (the primary source informing NeST thus far^37^) but, in the future, might incorporate information from complementary proteomics technologies such as proximity ligation^38^, size-exclusion chromatography^39,40^ or spatial imaging^41^. While pathway databases are sometimes treated as gold standards (especially literature-curated databases such as Gene Ontology and Reactome), knowledge of molecular pathways remains incomplete, particularly as it relates to specific tumor states and subtypes.

Using this platform, we identified a set of eight core assemblies for which genetic alterations are associated with anti-CDK4/6 response, seven of which were validated by one or more CRISPR screens (Figs. 4 and 5). These assemblies are not focused solely on cyclin-dependent control of the cell cycle (Fig. 3a). Nonetheless, ample literature support can be found for the involvement of many of these other assemblies in anti-CDK responses, such as those related to androgen receptor (AR) signaling^42^, EGF/fibroblast growth factor (FGF) signaling^43^, DNA damage response^44^ and the MDM2–p53 pathway^45^. Regarding the identification of an EGF/FGF signaling assembly, recent studies have found that the EGF receptors *EGFR* and *ERBB2* are associated with palbociclib response^46^ and that the genetic alteration status of *FGFR1/2* and their associated FGF ligands has promise as a marker of acquired resistance^43^. Furthermore, ongoing clinical trials are assessing the combination of anti-CDK4/6 treatments with insulin-like growth factor inhibition (trial no. NCT03099174) or with EGFR inhibition (trial no. NCT03065387) in various tumor types. In NeST-VNN, the EGF/FGF complex combines each of these alterations, which have largely been reported separately, into a single integrated effect including alterations in yet additional receptor tyrosine kinases (for example, ERBB3/4).

The model also highlights a notable role for NeST:85 (histone-mediated transcription regulation), which integrates both well-known and understudied factors. Treatment with CDK4/6 inhibitors induces chromatin structure remodeling mediated by histone acetyltransferases and histone deacetylases (HDACs), leading to the expression signatures of senescence and cell differentiation^3^. Accordingly, genetic alterations affecting proteins of the NeST:85 assembly, including the histone acetylases *CREBBP* and *EP300* (ref. 47), the HDACs *HDAC1* and *HDAC2* (ref. 48), and transcription factors such as *TP53* (ref. 49) and *MYC*^50^, have been previously documented to modulate the anti-CDK4/6 drug response. Using CRISPRa to model the effects of CNAs, we observed that increased expression of *KAT6A* and *TBL1XR1*, which are also components of the NeST:85 assembly, leads to increased S-phase entry (Fig. 5). *KAT6A*, also known as *MYST3*/*MOZ*, encodes a histone lysine acetyltransferase that is amplified in many cancer types^51^ (Fig. 5b). Relevant to the NeST:85 assembly, *KAT6A* has been previously documented to regulate cell-cycle arrest and differentiation through the transcription factors p53 (ref. 52) and RUNX1 (ref. 53); it is a frequent translocation partner of other assembly members such as EP300 and CREBBP^54^. TBL1XR1, also known as TBLR1, is an F-box-like protein involved in the recruitment of the ubiquitin conjugation system to histone modifier and transcriptional repression complexes^55,56^. Subsequent proteasomal degradation of these complexes is essential for transcriptional activation by *AR*, as captured by the NeST:85 assembly, as well as other transcription factors such as the estrogen receptor (*ER*)^57^. Notably, increases in *KAT6A* and *TBL1XR1* expression were associated with higher phosphorylation levels of RB, the central transcriptional repressor targeted by CDK cell-cycle control (Fig. 5g,h), suggesting that they may promote drug resistance by increasing the transcription, abundance or activity of the upstream CDK4–CDK6–cyclin D regulatory complex. The possible combination of HDAC inhibitor therapies with cell-cycle inhibitors has been previously proposed^48^; this study further underscores this potential and delineates alternative targets. Indeed, KAT6A inhibitors are under development and have demonstrated promising effectiveness for inducing cellular senescence^58–60^ (clinical trial NCT04606446). In the tumor cells characterized here (A549; Fig. 5), *TBL1XR1* has a T290A missense mutation of unknown significance whose impact will require further investigation.

In summary, the predictive models presented in this study build from and substantially develop the concept of an integrated response to therapy. In such an integrated response, diverse effects converge on biological machinery at multiple levels to produce an overall treatment outcome. This concept may explain the difficulty in identifying individual genetic biomarkers of palbociclib drug response. It also speaks to the challenge of patient-to-patient heterogeneity and illustrates one means by which knowledge of cellular machinery can be used to score a diverse population of cancer patients presenting unique patterns of mutational aberrations. Such an integrated model may provide a worthwhile asset in achieving improved outcomes for patients and in efforts to evaluate novel therapeutics to overcome resistance.

## Methods

### Drug response data for model training

Drug response data were retrieved from the GDSC and CTRP databases^24–27^, covering a total of 692,859 cell line–drug pairs comprising 1,244 cell lines and 888 drugs. The data from the two datasets were harmonized as follows. Drug information: each molecule’s published name, synonym or SMILES (Simplified Molecular Input Line Entry System) string was queried using PubChemPy. The associated InChIKey was extracted and used to identify duplicate drugs (within or between datasets). Cell viability data: for CTRP, the vehicle control-normalized average percent viability files were used. For GDSC1 and GDSC2, data were normalized to ‘cells-only’ and ‘dimethyl sulfoxide (DMSO) control’ wells, respectively, on a per-plate basis. Data were averaged across replicates within each dataset. For drug response measurement, we used AUC, in which AUC = 0 corresponds to complete cell killing and AUC = 1 corresponds to no cell killing; AUC > 1 represents a growth advantage conferred by the drug. AUCs calculated in this study agreed with AUCs reported by the original consortia (Pearson correlations of 0.92, 0.83, 0.91 and 0.91 for CTRP1, CTRP2, GDSC1 and GDSC2, respectively). For multiple AUCs for the same drug across different consortia, we used each replicate sample as a separate training instance. Genetic alteration data: a panel of 718 clinical genes was assembled from the union of genes assessed by FoundationOne CDx^20^, Tempus xT^21^, PALOMA-3 trial^61^ or Project GENIE^22^, each of which assesses mutations and/or copy number aberrations. To compile genotypes for all cell lines, we extracted nonsynonymous coding mutations and copy number alterations for the 718 clinical panel genes from the Cancer Cell Line Encyclopedia (CCLE, release 22Q1)^23^. Genes were marked as either mutated (‘1’) or unmutated (‘0’), with mutations filtered for the following types: missense/nonsense/nonstop mutations, frameshift insertions/deletions, splice site/region variations and in-frame insertions/deletions. Similarly, genes were marked as amplified (‘1’) or unamplified (‘0’) and deleted (‘1’) or undeleted (‘0’). Together, mutations, CNAs and CNDs served as features for each of the clinical panel genes. Of the 888 drugs available from the CCLE and/or GDSC, we selected the 51 drugs (palbociclib and 50 others) with the highest variation in the observed drug responses across cell lines (corresponding to s.d. ≥ 0.3).

### Structural architecture of the NeST-VNN model

Construction of the NeST hierarchy of cancer protein assemblies has been thoroughly detailed elsewhere^19^. Briefly, AP–MS protein interaction data for 61 known cancer proteins were integrated with a compendium of other systematically generated datasets informing protein–protein associations, including protein–protein interaction, mRNA coexpression, protein coexpression, genetic codependency and sequence similarity. Such integration resulted in a large network of approximately 1.8 × 10^8^ protein–protein interactions among 19,035 proteins. Multiscale community detection was performed to detect approximately 2,300 densely connected sets of proteins, herein called protein assemblies. Assemblies were nested (that is, organized hierarchically), with larger assemblies containing smaller ones, forming ‘parent–child’ assembly relations. This hierarchy has been used earlier^19^ to perform a comprehensive analysis of somatic coding mutations in The Cancer Genome Atlas (TCGA)^62^, identifying significant convergence of mutations on a set of 395 protein assemblies, named NeST^19^. Here, we filtered the NeST hierarchy to identify the subset of assemblies encoded by at least five genes represented on the 718-gene clinical panel, producing a final hierarchy of 131 assemblies distributed over seven layers.

### Model training

The filtered NeST hierarchy was used to embed a deep neural network for drug response prediction, which we refer to as NeST-VNN (Extended Data Fig. 1a). We define an m×3 input matrix as I, where $I_{i,j}\in \{0,\;1\}$, with m denoting the number of genes and 3 the number of gene alteration types (mutation, CNA and CND). For any input sample (tumor cell line, PDX or patient tumor), somatic genetic alterations for each gene and type are marked by 1 and otherwise 0. The first layer in NeST-VNN converts these input features to gene-level representations, $I_{g}\in R^{m}$, as follows:

$$\left.\left.I_{g}=\;\text{BatchNorm}(\text{tanh}(\text{Linear}((I)\right)\right)$$

‘BatchNorm’ indicates batch normalization^63^; ‘tanh’ indicates a hyperbolic tangent function; and ‘Linear’ indicates a linear transformation. Here, the linear transformation is applied for each row in I so that the three gene alteration values for each gene are converted into a single value. The remaining seven layers of NeST-VNN follow the structure of the NeST protein assembly hierarchy, where each assembly is represented by some number of neurons N, a hyperparameter. A dropout^64^ of 0.3 (selected through hyperparameter optimization) was added to the last four layers. Assembly state is defined as a function of the states of its K child assemblies and M additional genes (genes for which the protein products are not present in any descendant assemblies). Denoting an assembly input vector as $I_{s}$ and an output vector as $O_{\text{s}}$, we have

$$O_{s}=BatchNorm\left(tanh\left(Linear\left(Dropout\left(I_{s}\right)\right)\right)\right)$$

Here, $I_{s}$ has dimension N×(N×K+M) and $O_{s}$ has dimension N. We define ‘in silico activity’, a representative singular value for assembly state, as the first principal component^65^. The NeST-VNN objective function (Loss) aggregates the mean squared error (MSE) across every assembly in the hierarchy:

$$Loss=MSE\left(Linear\left(O_{\text{root}}\right),y\right)+\alpha \sum_{s\neq \text{root}\;}\;MSE\left(Linear\left(O_{s}\right),y\right)+\beta ∥W∥$$

The parameter α was set to 0.3; β is a tuned hyperparameter. y represents the actual AUC.’Linear’ denotes the linear function used for transforming the vector $O_{i}$ to a scalar. W denotes the weights of the neural network. Weight optimization was performed using AdamW^66^.

### Model benchmarking

For baseline benchmarking, we trained the RF^67^, ElasticNet^68^ and black-box ANN^69^ (allotted the same number of neurons and layers as the NeST-VNN model) models using the Python scikit-learn package^70^. For all models, including NeST-VNN, we used nested fivefold cross-validation^71^, producing five models for each drug. For each fold setting, we split 64% of cell lines as a training set, 16% as a validation set (used for hyperparameter tuning) and 20% as a test set, ensuring that cell line replicate measurements (for example, from different datasets) were not split between the test and training sets. Hyperparameters were optimized with Optuna^72^. NeST-VNN was implemented in PyTorch and trained using five GPU (graphics processing unit) servers containing four NVIDIA Tesla V100s, each with 5,120 CUDA (Compute Unified Device Architecture) cores and 32-GB GDDR6 random access memory. All five NeST-VNN models were evaluated in downstream analyses.

### Translation to cancer patients

Data from the American Association for Cancer Research Project GENIE metastatic breast cohort^22^ were used to validate the performance of the NeST-VNN model in retrospective clinical application. We extracted nonsynonymous coding mutations, CNAs and CNDs across 360 genes for 226 patients with ER^+^, HER2^−^ metastatic breast cancer along with their overall survival (months) and censorship information. We did not consider gender or sex. Of these patients, 67 had been treated with CDK4/6i plus endocrine therapy. The remaining 159 patients were treated with endocrine therapy alone. Patients were excluded if they had been treated with additional targeted therapies, such as mammalian target of rapamycin (mTOR) or AKT inhibitors. Tumor genomic data were converted to calls (0 = unaltered, 1 = altered) for all gene mutation, CNA and CND features. Features used by NeST-VNN that were not assessed in the clinical trial were represented as unaltered. We predicted patient response to CDK4/6 inhibition using the average AUC over the five pretrained palbociclib models and then thresholded this value as described in the main text (Fig. 2d). Patients whose status label was ‘living’ at 120 months were censored.

### Translation to PDXs

We analyzed a PDX dataset^28^, which contained 172 tumor samples treated with a CDK4/6i (ribociclib) across five tumor types (breast carcinoma, non-small cell lung carcinoma, cutaneous melanoma, colorectal cancer and pancreatic ductal carcinoma). Treatment responses had been measured by changes in the volume of the tumor xenograft over time, with an accompanying determination of treatment time and a classification according to the RECIST (Response Evaluation Criteria in Solid Tumors) standard (including categories of progressive disease, stable disease, partial response and complete response). PDX samples had been genomically characterized, covering 660 of the 718 genes in the NeST-VNN gene set. Similar to the procedure for cell lines and patients, tumor genomic data were converted to calls (0 = unaltered, 1 = altered) for all gene mutation, CNA and CND features. Features used by NeST-VNN that were not assessed in the PDX data were represented as unaltered. We predicted the responses of PDX tumors to CDK4/6 inhibition as the average AUC over the five pretrained NeST-VNN models for palbociclib and then thresholded this score as described in the main text (Fig. 2c).

### Model dependence on the number of genes used for prediction

**Extended Data Fig. 5 |:**
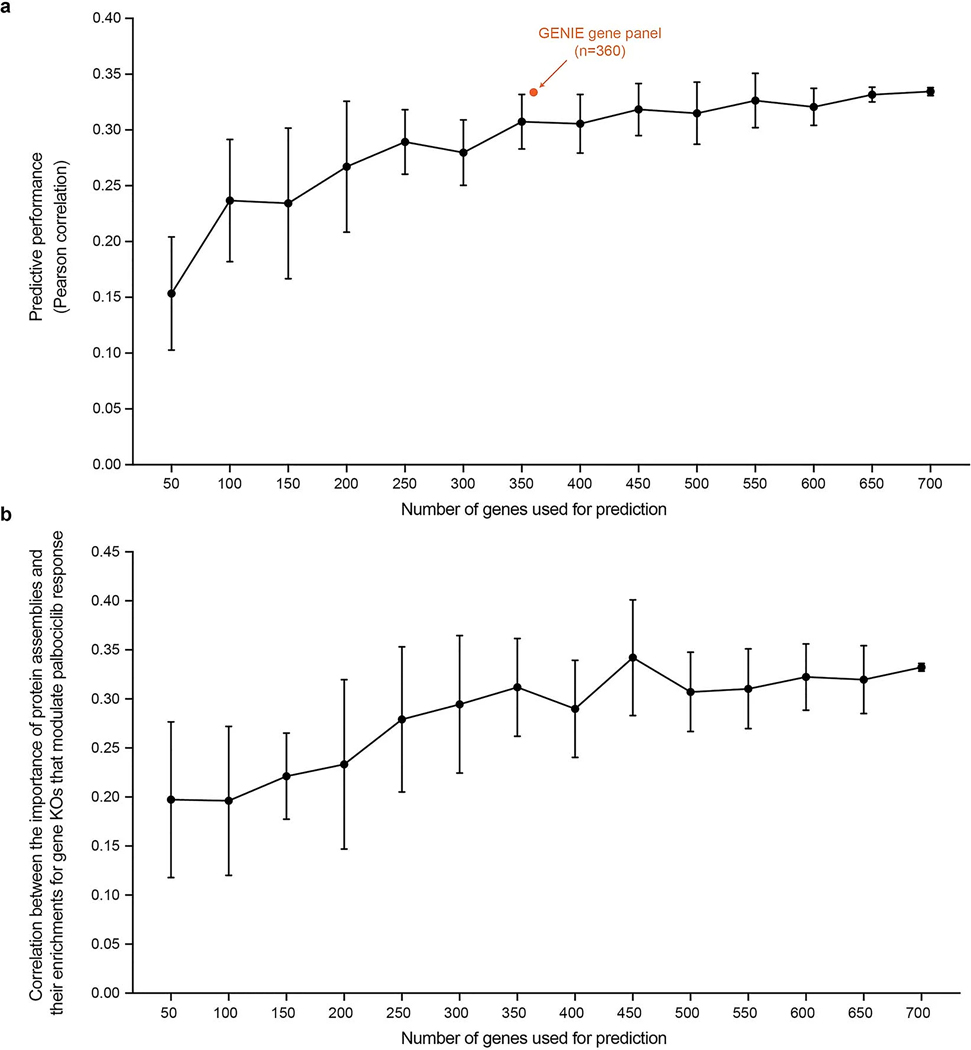
Supplemental analysis of NeST-VNN robustness. **a**, Predictive performance of NeST-VNN according to the number of genes used for prediction. Predictive performance is defined by the Pearson correlation between the predicted and actual drug responses. Each point represents the average predictive performance (y-axis) from 10 repeated experiments, with each experiment drawing a different random selection of genes of a given set size (x-axis). The error bar indicates the standard deviation of the predictive performance across these experiments. The orange point indicates the predictive performance (Pearson ρ = 0.33) using the GENIE gene panel (n = 360) for prediction. **b**, Correlation (Pearson ρ) between the importance of protein assemblies for model prediction and their enrichments for gene KOs that modulate palbociclib response (y-axis) as a function of the number of genes used for prediction (x-axis). Each point represents the average Pearson correlation from 10 repeated experiments, with each experiment drawing a different random selection of genes of a given set size. The error bar indicates the standard deviation of the Pearson correlation across these experiments.

Given the difference in the number of genes used for prediction in cell lines (*n* = 718) versus GENIE analysis (*n* = 360) or PDX analysis (*n* = 660), we systematically studied the dependence of model performance on the number of genes for which genetic alteration data are provided. We computed the average predictive performance of the pretrained NeST-VNN model when it is supplied with data for diminishing numbers of genes (Extended Data Fig. 5a). We found that, at a gene set size of 350 (similar to the number of genes characterized in the GENIE study), the average performance is only slightly less than that obtained when using all genes (ρ = 0.30 versus ρ = 0.33), with a more precipitous fall in performance seen for 200 genes or fewer. A similar pattern was observed when we compared the assembly importance scores with their enrichments for gene KOs that modulate the response to palbociclib treatment (Extended Data Fig. 5b). Notably, we also found that the precise panel of genes used by GENIE performs better than expected compared to a random subsampling (Extended Data Fig. 5).

### Identifying important assemblies and genes (model interpretation)

To determine which assemblies were important for drug response prediction in cell lines, PDX or clinical samples, we adopted a variation of the ‘relative local improvement in predictive power’ method as previously reported^13^. Each assembly was modeled using linear regression, with the aim of evaluating how well its NeST-VNN neuron values capture the NeST-VNN overall drug response prediction. Each assembly k was assigned a g×N matrix $P_{k}$, where g is the number of samples and N is the number of neurons. $P_{k}$ was then used in a linear ridge regression^73^ model to predict the NeST-VNN drug response D, creating models $M_{1},M_{2},\ldots ,M_{k}$. The following function was minimized for each model:

$${min}_{w}\;{∥P_{k}w-D∥}_{2}^{2}+\alpha ∥w{∥}_{2}^{2}$$

where w is a vector of the coefficients of length N and α imposes an L2 penalty on coefficient complexity. Assembly ‘importance’ (Fig. 3 and Extended Data Figs. 3 and 5) is the Spearman correlation (ρ) between $M_{k}$ and D. The mean correlation of the five NeST-VNN models was reported. A higher score indicates an assembly whose neuron values contributed more strongly to NeST-VNN predictions and can, therefore, be considered important. To assess statistical significance, we generated a null distribution of assembly importance scores, as follows. We randomly rearranged gene assembly memberships in the NeST-VNN while preserving the assembly size and parent–child relationships. We trained 500 null models with these random rearrangements and calculated assembly importance for each null. One-tailed *t* tests were used to evaluate whether the assembly importance scores from the five NeST-VNN models were greater than the assembly importance scores from the nulls, with a Benjamini–Hochberg control for false discovery rate (FDR; Fig. 3a). Finally, we defined ‘core assemblies’ as those with an importance score of ≥0.5 and an FDR of ≤0.1, while excluding less important redundant assemblies (Jaccard similarity > 0.5). To identify specific genetic alterations in the NeST:85 assembly associated with palbociclib resistance (Fig. 5c), we performed L1-norm regularized logistic regression^74,75^. Genetic alterations (mutations, CNAs, CNDs) for the 15 assembly genes were used as regression features to predict AUCs. AUC values in the top 30% were encoded as 1 to represent resistance, whereas AUC values in the bottom 30% were encoded as 0 to represent sensitivity. Nonzero coefficients from the fitted model were recognized as important alterations governing drug response, with the sign indicating whether the presence of alterations contributed to resistance (plus) or sensitivity (minus). We used scikit-learn^70^ with logistic regression settings of penalty = ‘l1’, *C* = 0.01 (default for other parameters).

### Comparison of the interpretability of NeST-VNN and RF

**Extended Data Fig. 6 |:**
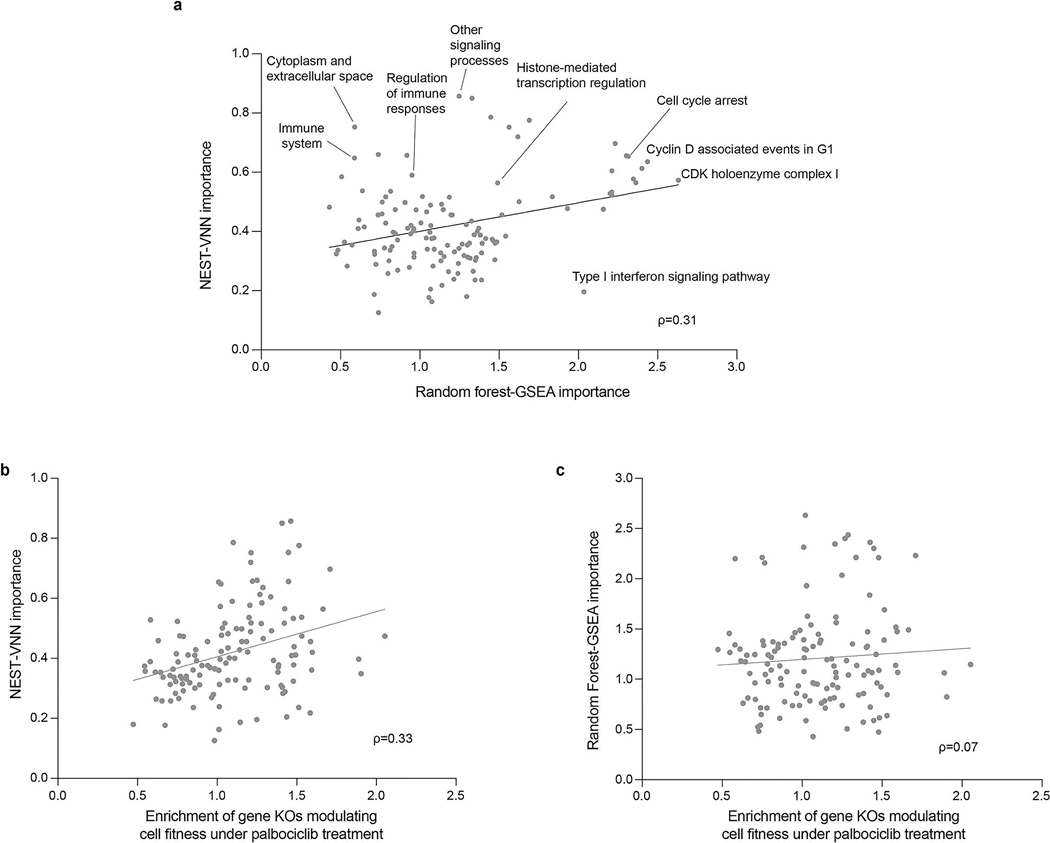
Supplemental comparison of NeST-VNN versus Random Forest models. **a**, Scatter plot of assembly importances from a Random forest-GSEA approach (x-axis) versus a NeST-VNN approach (y-axis). **b**, Scatter plot of assembly importance in the NeST-VNN model (y-axis) versus enrichment of gene KOs modulating cell fitness under palbociclib treatment (x-axis). Each dot represents an assembly (n = 130). Rho (ρ) indicates the Pearson correlation. **c**, Same as panel **b** except the y-axis indicating Random Forest-GSEA importance.

We systematically evaluated the assembly importance scores provided by NeST-VNN versus RFs^67^ using the genome-wide loss-of-function screen for palbociclib treatment. To determine the assembly importance score for the RF models, we performed gene set enrichment analysis (GSEA^76^, implemented using GSEApy^77^) on the gene list ranked according to the gene-level feature importance scores derived from the RF models. The absolute normalized enrichment scores generated from GSEA were used as assembly importance scores for the RF models. Assembly importances in the NeST-VNN versus RF models were moderately but not completely correlated (ρ = 0.31; Extended Data Fig. 6a). Relevant to the differences, we found that the NeST-VNN importance of an assembly was also moderately correlated with its enrichment for gene KOs conferring palbociclib sensitivity or resistance (ρ=0.33; Extended Data Fig. 6b); in contrast, RF assembly importance showed a correlation that was substantially weaker (ρ=0.07; Extended Data Fig. 6c). Thus, while RF models can achieve comparable predictive performance by identifying individual gene mutations that are indicative of drug response (Extended Data Fig. 2a,c), NeST-VNN demonstrates its strength by integrating the effects of such mutations within predictive cancer protein assemblies.

### Genome-wide CRISPR KO chemogenetic screen

Core protein assemblies were validated using a genome-wide CRISPR–Cas9 screen in MCF7 cells exposed to palbociclib treatment^33^ (Fig. 4a) (Gene Expression Omnibus accession no. GSE192525). This screen had been run previously using the GeCKO (genome-wide CRISPR KO) v2 library. Gene-level *z* scores (referred to as ‘normZ’) from that study were used to indicate the effects of gene KO on cell fitness in the context of CDK4/6 inhibition (Fig. 4b). As a reference, the cell fitnesses of gene KOs (provided as Chronos scores^78^) in the MCF7 cell line in the absence of CDK4/6i treatment (Fig. 4c) were obtained from the DepMap (Dependency Map) project^34^ (https://depmap.org/portal/).

### Dual CRISPR KO combinatorial screen

The genome-wide chemogenetic data (above section) were complemented by a de novo dual CRISPR screen performed in-house in MCF7 (HTB-22), MCF10A (CRL-10317) and MDAMB231 (CRM-HTB-26) cell lines from American Type Culture Collection (ATCC) (Fig. 4a). Cells were grown in DMEM with 10% FBS, screened for *Mycoplasma* contamination by PCR and verified by short tandem repeat (STR) testing (IDEXX BioAnalytics). CRISPR–Cas9 nuclease was stably integrated by a lentivirus. LentiCas9-Blast (Addgene plasmid no. 52962) and lentiCRISPR v2 (Addgene plasmid no. 52961) were gifts from F. Zhang^79^. Blasticidin was used to select Cas9 stable integrants. Cas9 protein expression was confirmed by capillary western blot analysis (Wes, ProteinSimple). We constructed a library of double gRNA constructs targeting druggable targets (such as CDK4 and CDK6), tumor suppressors and oncogenes. Here, we analyzed a subset of data from individual genes from core assemblies (sgRNA1) together with *CDK4* or *CDK6* (sgRNA2) (Supplementary Table 3). The library was packaged into lentiviruses, and cells were infected to achieve a multiplicity of infection of 0.3. Puromycin (2.5 mg ml^−1^) selection was started 2 days after transduction. Selection continued for 7 days, after which puromycin was removed for the remainder of the screen. Cells were maintained in exponential growth by isolating and removing a fraction of cells every 2–3 days. We analyzed data from two time points at approximately 14 and 21 days. DNA was extracted from cells with a Blood and Cell Culture DNA Mini kit (Qiagen). To assess the relative frequencies of gRNAs before and after selection, we amplified gRNA sequences from genomic DNA by PCR and prepared them for HiSeq 4000 sequencing (Illumina). Standard Illumina primers were used for library preparation, and 100-bp paired-end reads were collected. Data quality was assessed with FastQC. The fitness effects of gene KOs at a time point were determined as the fold enrichment of a construct compared to the relative abundance of that construct in the plasmid library. Fitness measurements were normalized to the median fitness for nontargeting guides. The mean *z* score across two biological replicates, two time points and genes in each assembly was then determined and plotted (Fig. 4f,g).

### Production of a dCas9-expressing stable cell line

CRISPRa experiments were performed in A549 cells stably expressing dCas9 together with the VPR transcriptional activation complex. For this purpose, 293T cells (CRL-3216, ATCC) were cotransfected with a second-generation packaging plasmid (pCMV-dR8.2, Addgene 8455), vesicular stomatitis virus-G envelope-expressing plasmid (pMD2.G, Addgene 12259) and dCas9–VPR lentiviral plasmid (hCMV-Blast-dCas9-VPR, Horizon Discovery) using Lipofectamine 3000 (Invitrogen, L3000015). Viral supernatant was collected and cleared of cell debris by centrifugation and Steriflip column (Millipore, SE1M003M00). Lentivirus was concentrated using Amicon Ultra-15 centrifugal filters (Millipore, Z706345). Viral titer was determined through serial dilution. Subsequently, A549 cells (CCL-185, ATCC) were grown in a virus-containing medium (DMEM/F12: 10% FBS, 100 IU ml^−1^ penicillin/streptomycin) with 8 μg ml^−1^ polybrene for 72 h, followed by medium washout and selection with blasticidin (3.5 μg ml^−1^) for 6 days. After selection, cells were cultured with maintenance-dose blasticidin (0.35 μg ml^−1^) every other passage. The identity of stable dCas9 A549 cells was confirmed by STR testing (IDEXX BioAnalytics, August 31, 2020).

### CRISPRa screen

A custom panel of sgRNA expression plasmids targeting genes in the NeST:85 assembly was obtained from Horizon Discovery (Fig. 5 and Supplementary Table 4). Controls included an NTC sgRNA and an overexpression (positive control) sgRNA targeting OCT4 (not a component of NeST:85). dCas9–VPR stable A549 cells were plated in a complete medium and transfected the next day with sgRNA plasmids for 24 h using FuGENE HD (Promega). Cells were selected with puromycin (0.44 μg ml^−1^) for 48 h and then lifted onto appropriate plates for further experimentation, where they were permitted to recover for 72 h. RNA was collected using the TRIzol reagent (Invitrogen, 15596026) and the RNeasy Mini kit (Qiagen, 74104). cDNA was synthesized using the iScript cDNA kit (Bio-Rad, 1708891). qPCR was performed using SYBR green, and cycle threshold (*C*_t_) values were compared for genes overexpressed by CRISPRa versus NTC samples (Extended Data Fig. 4c and Supplementary Table 4).

### EdU assays for S-phase entry

Transfected cells were plated in collagen-coated glass-bottom 96-well plates in a complete medium containing palbociclib (4 μM) for 24 h. Components of EdU Click-iT (Thermo Fisher Scientific, C10337) were prepared as instructed. Cells were labeled for 4 h with 10 μM EdU-labeling solution in the medium and then counterstained with Hoechst dye (1:10,000) for 10 min. Cells were fixed in 3.75% formaldehyde for 10 min at room temperature and then washed, permeabilized and stained according to the manufacturer’s instructions. Images were collected using a Keyence microscope (BZ-X800) fitted with a 4× objective and green fluorescent protein/fluorescein isothiocyanate (Chroma, C209879) and DAPI (Chroma, C209877) filters. Images were processed in bulk using scikit-image^80^. Cells were identified using Hoechst counterstain and then assessed for EdU incorporation (Fig. 5e,f).

### Capillary western assays for RB status

Transfected cells were treated with palbociclib for 24 h and then trypsinized and washed in cold PBS; pellets were frozen at −80 °C. Protein was extracted in a hot 1× MES SDS running buffer (Invitrogen, NP0002) for 10 min. Cooled samples were vortexed for 2 min with glass beads (Sigma, G8772). cOmplete EDTA-free protease inhibitor cocktail (Roche, 04693132001) and PhosSTOP (Roche, 4906845001) were added to the cleared lysate. Protein was quantified using the Pierce 660-nm protein assay reagent (Thermo Fisher Scientific, 22662). Protein analysis was performed on a capillary-based western blot system (Wes, ProteinSimple, product no. 004–600) according to the manufacturer’s instructions using the 12- to 230-kDa separation module (ProteinSimple, SM-W001) and either the anti-rabbit detection module (ProteinSimple, DM-001) or the anti-mouse detection module (ProteinSimple, DM-002). Protein samples were diluted to 1 μg ml^−1^ in 0.1× sample buffer (ProteinSimple, 042–195) and then mixed with fluorescent master mix and heated at 95 °C for 5 min. Anti-phospho-RB Ser807/811 (mouse monoclonal antibody, clone D20B12, 1:100, Cell Signaling, 8516) or anti-actin (rabbit polyclonal antibody, 2 μM, Novus, NB600–532) was used as the primary antibody, whereas a horseradish peroxidase-conjugated anti-rabbit antibody (ProteinSimple, DM-001) was used as a secondary antibody. Program settings were as follows: separation at 375 V, 25 min; blocking reagent, 15 min; 20-s wash (for runs with phospho-RB only); primary antibody blocking, 35 min; two 150-s washes; secondary antibody blocking, 35 min; 150-s wash; chemiluminescence detection, from 1 to 512 s. Electropherograms (Fig. 5g,h) were inspected to check whether automatic peak detection required manual correction.

### Statistics and reproducibility

All wet laboratory experiments were performed in biological duplicates with three to four technical replicates. No statistical method was used to predetermine sample sizes. The experiments were not randomized. The investigators were not blinded to allocation during experiments or outcome assessments. EdU assays were evaluated computationally with data-quality threshold filters as described above. For the survival analysis, patients were excluded if they had been documented to receive a targeted therapy other than a CDK4/6i (that is, an mTOR or AKT inhibitor), as these other targeted therapies were not the focus of our study. Statistical tests were performed as described in each section assuming data were normally distributed where appropriate, but this was not formally tested.

## Extended Data

## Supplementary Material

Supplementary Tables 1-4

## Data Availability

The datasets used in this study are all publicly available. GDSC version 1: https://www.cancerrxgene.org/downloads/bulk_download; GDSC version 2: https://www.cancerrxgene.org/downloads/bulk_download; CTRP version 1: https://portals.broadinstitute.org/ctrp.v1/; CTRP version 2: https://portals.broadinstitute.org/ctrp.v2.1/; DepMap 22Q1: https://doi.org/10.6084/m9.figshare.19139906.v1; PDX,: https://www.nature.com/articles/nm.3954; Project GENIE: https://genie.cbioportal.org/study/summary?id=brca_akt1_genie_2019; genome-wide CRISPR KO chemogenetic screen: https://www.ncbi.nlm.nih.gov/geo/query/acc.cgi?acc=GSE192525 (Gene Expression Omnibus accession no. GSE192525). The Cytoscape session containing the NeST-VNN hierarchy and the pretrained models are available on GitHub. Cytoscape session: https://github.com/idekerlab/nest_vnn/blob/main/misc/NeST_VNN_Palbociclib.cys; pretrained models: https://github.com/idekerlab/nest_vnn/tree/main/pretrained_models/palbociclib. Source data are provided with this paper.
