# Supplementary material for: A deep learning model of tumor cell architecture elucidates response and resistance to CDK4/6 inhibitors
Source: Nat Cancer. 2024 Mar 5;5(7):996–1009. doi: 10.1038/s43018-024-00740-1 (PMC11286358; doi:10.1038/s43018-024-00740-1)
Supplement: Supplementary file 2 — Reporting Summary [file 43018_2024_740_MOESM2_ESM.pdf]

Corresponding author(s): Trey Ideker
Last updated by author(s): 12/17/2023

Reporting Summary

Nature Portfolio wishes to improve the reproducibility of the work that we publish. This form provides structure for consistency and transparency in reporting. For further information on Nature Portfolio policies, see our Editorial Policies and the Editorial Policy Checklist.

Statistics

For all statistical analyses, confirm that the following items are present in the figure legend, table legend, main text, or Methods section.

n/a	Confirmed	
☐	☒	The exact sample size (n) for each experimental group/condition, given as a discrete number and unit of measurement
☐	☒	A statement on whether measurements were taken from distinct samples or whether the same sample was measured repeatedly
☐	☒	The statistical test(s) used AND whether they are one- or two-sided<br>*Only common tests should be described solely by name; describe more complex techniques in the Methods section.*
☒	☐	A description of all covariates tested
☐	☒	A description of any assumptions or corrections, such as tests of normality and adjustment for multiple comparisons
☐	☒	A full description of the statistical parameters including central tendency (e.g. means) or other basic estimates (e.g. regression coefficient) AND variation (e.g. standard deviation) or associated estimates of uncertainty (e.g. confidence intervals)
☐	☒	For null hypothesis testing, the test statistic (e.g. F, t, r) with confidence intervals, effect sizes, degrees of freedom and P value noted<br>*Give P values as exact values whenever suitable.*
☒	☐	For Bayesian analysis, information on the choice of priors and Markov chain Monte Carlo settings
☒	☐	For hierarchical and complex designs, identification of the appropriate level for tests and full reporting of outcomes
☐	☒	Estimates of effect sizes (e.g. Cohen's d, Pearson's r), indicating how they were calculated

Our web collection on statistics for biologists contains articles on many of the points above.

Software and code

Policy information about availability of computer code

Data collection	Python 3.8, PyTorch 1.8, Cuda 11.1
Data analysis	Optuna, Scikit-learn, Scikit-image.<br>The software implementation of NeST-VNN is available at https://github.com/idekerlab/nest_vnn

For manuscripts utilizing custom algorithms or software that are central to the research but not yet described in published literature, software must be made available to editors and reviewers. We strongly encourage code deposition in a community repository (e.g. GitHub). See the Nature Portfolio guidelines for submitting code & software for further information.

Data

Policy information about availability of data

All manuscripts must include a data availability statement. This statement should provide the following information, where applicable:

- Accession codes, unique identifiers, or web links for publicly available datasets
- A description of any restrictions on data availability
- For clinical datasets or third party data, please ensure that the statement adheres to our policy

The datasets used in this study are all publicly available.
GDSC version 1: https://www.cancerrxgene.org/downloads/bulk_download
GDSC version 2: https://www.cancerrxgene.org/downloads/bulk_download

CTRP version 1: https://portals.broadinstitute.org/ctrp.v1/
CTRP version 2: https://portals.broadinstitute.org/ctrp.v2.1/
DepMap 22Q1: https://doi.org/10.6084/m9.figshare.19139906.v1
PDX: https://www.nature.com/articles/nm.3954
Project GENIE: https://genie.cbioportal.org/study/summary?id=brca_akt1_genie_2019
Genome-wide CRISPR KO chemogenetic screen: https://www.ncbi.nlm.nih.gov/geo/query/acc.cgi?acc=GSE192525 (GEO accession number: GSE192525).

The Cytoscape session containing the NeST-VNN hierarchy and the pre-trained models are available on GitHub.
Cytoscape session: https://github.com/idekerlab/nest_vnn/blob/main/misc/NeST_VNN_Palbociclib.cys
pre-trained models: https://github.com/idekerlab/nest_vnn/tree/main/pretrained_models/palbociclib

Research involving human participants, their data, or biological material

Policy information about studies with human participants or human data. See also policy information about sex, gender (identity/presentation), and sexual orientation and race, ethnicity and racism.

Reporting on sex and gender	N/A
Reporting on race, ethnicity, or other socially relevant groupings	N/A
Population characteristics	N/A
Recruitment	N/A
Ethics oversight	N/A

Note that full information on the approval of the study protocol must also be provided in the manuscript.

Field-specific reporting

Please select the one below that is the best fit for your research. If you are not sure, read the appropriate sections before making your selection.

☒ Life sciences ☐ Behavioural & social sciences ☐ Ecological, evolutionary & environmental sciences

For a reference copy of the document with all sections, see nature.com/documents/nr-reporting-summary-flat.pdf

Life sciences study design

All studies must disclose on these points even when the disclosure is negative.

Sample size	We considered 1244 cell lines from the CCLE database, 888 drugs from CTRP and GDSC databases, and 428 patient samples from project. GENIE. These published datasets were considered in their entirety and samples were excluded as described below. For wet lab experiments, no statistical method was used to predetermine sample size.
Data exclusions	Patients were excluded from survival analysis if they had been treated with another targeted inhibitor drug (i.e. anti-AKT or anti-MTOR agents), as these drugs were not the main subject of the current study.
Replication	All wet lab experiments were performed in biological duplicate with three to four technical replicates.
Randomization	For five-fold cross-validation, we randomly divided cell lines into five equal parts while ensuring that duplicate cell lines or drugs were present only in a single fold.
Blinding	There is no blinding in this study.

Reporting for specific materials, systems and methods

We require information from authors about some types of materials, experimental systems and methods used in many studies. Here, indicate whether each material, system or method listed is relevant to your study. If you are not sure if a list item applies to your research, read the appropriate section before selecting a response.

Materials & experimental systems

n/a	Involved in the study
☐	☒ Antibodies
☐	☒ Eukaryotic cell lines
☒	☐ Palaeontology and archaeology
☒	☐ Animals and other organisms
☒	☐ Clinical data
☒	☐ Dual use research of concern
☒	☐ Plants

Methods

n/a	Involved in the study
☒	☐ ChIP-seq
☒	☐ Flow cytometry
☒	☐ MRI-based neuroimaging

Antibodies

Antibodies used

anti-phospho-RB Ser807/811 (Mouse mAb clone D20B12, 1:100, Cell Signaling, #8516), anti-actin (rabbit polyclonal, 2µM, Novus, NB600-532), HRP-conjugated secondary antibodies (ProteinSimple DM-002)

Validation

Anti-phospho-RB antibody was validated in-house by treating samples with CDK4/6 inhibitor and observing the expected decrease in band intensity.

The anti-actin antibody has >50 publications and is confirmed compatible with ProteinSimple (https://www.novusbio.com/products/beta-actin-antibody_nb600-532).

Validation Statement from Cell Signaling:
To ensure our antibodies will work in your experiment, we adhere to the Hallmarks of Antibody Validation™, six complementary strategies that can be used to determine the functionality, specificity, and sensitivity of an antibody in any given assay. CST adapted the work by Uhlen, et. al., ("A Proposal for Validation of Antibodies." Nature Methods (2016)) to build the Hallmarks of Antibody Validation, based on our decades of experience as an antibody manufacturer and our dedication to reproducible science."

Eukaryotic cell lines

Policy information about cell lines and Sex and Gender in Research

Cell line source(s)

MCF7 (HTB-22 ATCC), MCF10A (CRL-10317, ATCC), MDAMB231 (CRM-HTB-26, ATCC), 293T (ATCC CRL-3216), A549 (ATCC CCL-185)

Authentication

All cell lines are confirmed by STR Testing (IDEXX Bioanalytics)

Mycoplasma contamination

All cell lines are confirmed negative for mycoplasma by PCR testing

Commonly misidentified lines
(See ICLAC register)

MCF7 are a widely used breast carcinoma cell line and were selected here as an additional breast cancer cell line.

Plants

Seed stocks

N/A

Novel plant genotypes

N/A

Authentication

N/A
